# A robot-assisted acoustofluidic end effector

**DOI:** 10.1038/s41467-022-34167-y

**Published:** 2022-10-26

**Authors:** Jan Durrer, Prajwal Agrawal, Ali Ozgul, Stephan C. F. Neuhauss, Nitesh Nama, Daniel Ahmed

**Affiliations:** 1 Acoustic Robotics Systems Lab, Institute or Robotics and Intelligent Systems, Department of Mechanical and Process Engineering, ETH Zurich, Zurich, Switzerland; 2 Department of Molecular Life Sciences, University of Zurich, Zurich, Switzerland; 3 Department of Mechanical & Materials Engineering, University of Nebraska-Lincoln, Lincoln, NE USA

**Keywords:** Mechanical engineering, Acoustics

## Abstract

Liquid manipulation is the foundation of most laboratory processes. For macroscale liquid handling, both do-it-yourself and commercial robotic systems are available; however, for microscale, reagents are expensive and sample preparation is difficult. Over the last decade, lab-on-a-chip (LOC) systems have come to serve for microscale liquid manipulation; however, lacking automation and multi-functionality. Despite their potential synergies, each has grown separately and no suitable interface yet exists to link macro-level robotics with micro-level LOC or microfluidic devices. Here, we present a robot-assisted acoustofluidic end effector (RAEE) system, comprising a robotic arm and an acoustofluidic end effector, that combines robotics and microfluidic functionalities. We further carried out fluid pumping, particle and zebrafish embryo trapping, and mobile mixing of complex viscous liquids. Finally, we pre-programmed the RAEE to perform automated mixing of viscous liquids in well plates, illustrating its versatility for the automatic execution of chemical processes.

## Abstract (web summary)

Lab-on-a-chip systems have been widely used in microscale liquid manipulation and greatly benefit from automation. Durrer et al. show a robot-assisted acoustofluidic end effector system, comprising a robotic arm and an acoustofluidic device, that combines both robotic and microfluidic functionalities.

## Introduction

Liquid manipulation is the foundation of laboratory processes that are widely used throughout chemistry and biology research, the pharmaceutical and cosmetic industries, diagnostic centres, and clinical laboratories. Numerous applications such as polymerase chain reaction (PCR), immunoassays, combinatorial assays, drug screening and discovery, and genomic research require repetitive sample preparation steps, typically involving micro pipetting by manual operators^1–4^. These procedures, prone to human error and often time-consuming, have become expensive in terms of skilled personnel, operating cost, and reagent use^5–8^. In fact, sample preparation has become a significant bottleneck for experiments, particularly in genomic research and clinical laboratories; consequently, an increasing number of laboratories are considering realizing a more cost-efficient and effective workflow through robotics and automation^6^. Currently, there exist several do-it-yourself (DIY) and commercial robotics systems for liquid handling, particularly at the macroscale^9–14^. While the future of laboratory robotics is very exciting, with great potential in the combination of computer vision, machine learning, and artificial intelligence, most extant robotics technologies predominantly perform automated pipetting for handling liquids and furthermore primarily focus on mimicking the function of a human operator, such as in opening a vial, handling solid and liquid specimens, etc^5,6,15–19^. Many still consume large quantities of expensive biochemical reagents during their operation and lack the capacity for sample preparation at the microscale. Consequently, the development of an effective robotic platform to assist and augment common laboratory functionalities remains a work in progress.

To address this unmet need, recent research has proposed the use of an end effector, which is tethered to a robotic arm and can mimic certain functionality of a human hand to execute specific tasks with high precision in a repeatable manner. Current strategies include electrostatic^20–23^, magnetic^24,25^, and the widely-popular pneumatically controlled soft end effectors^16,20,21^, which are typically designed for grasping or manipulating macroscale objects measuring millimetres to centimetres in size. As of now, very little has been achieved in the area of effectively manipulating microscale objects with a robotic arm^6,15,26–29^. In contrast, for micromanipulation in general, ultrasound-based techniques leveraging several physical principles have been developed to trap and manipulate particles in the micro- or millimetre size range. For example, devices have been developed to trap microparticles at the pressure nodes of standing bulk^30^ and surface acoustic waves^31^, as well as at the nodes of Chladni’s plate^32^ and on the surfaces of oscillating sharp edges^33^, ciliary arrays^17^, and microbubbles^34,35^. However, these methods operate within a limited working environment and lack the flexibility offered by a robotic arm. Consequently, the combination of an acoustic field with a robotic arm has the potential to leverage acoustic-based fluid and particle manipulation techniques, while simultaneously offering unprecedented flexibility, yet acoustic-based end effectors have received little attention to date^6,15,26–29^.

Furthermore, trapping of microparticles using an external acoustic wavefield is typically static; when particles are to be dynamically manipulated, the resonance frequency of the wavefield must be adjusted manually. Such acoustic fields are generated with commercially-available piezo transducers, which have limited bandwidth, and so the acoustic microtraps can only be operated over limited distances^18,36^. Additionally, three-dimensional (3D) manipulation^4,37^ and selective trapping^38,39^ and manipulation of microparticles^40,41^ via acoustic methods have all proven extremely difficult to implement. Dynamic manipulation in a large working environment has recently been achieved by connecting acoustic devices to a manipulation stage. For example, a novel acoustic trapping device was implemented by depositing spiral electrodes on a piezoelectric substrate to pick and manoeuvre single cells using tightly focused radiation force^42^. In another endeavour, a multi-layered piezo actuator was connected to a micropipette, which produced a whirling flow able to trap and rotate a cell spheroid^29^. Notably, while these recent acoustic devices employed micromanipulators, the combination of acoustics and robotics for diverse laboratory functions (e.g., fluid pumping, particle trapping, droplet merging, etc.) is yet to be demonstrated^6^. Robotic arms offer a variety of unique advantages, including more than five axes of motion, the ability to programme complex paths, real-time motion planning, automation, repeatability, and the ability to operate in considerably broader working volumes.

Although microfluidics technologies offer great precision, control, and manipulation of liquid at the microscale, there is a pressing need for automation and multifunctionalities to advance the field considerably. In addition to enacting automation capabilities, the combination of robotics and microfluidics can also facilitate exciting new applications and multifunctionalities. Despite the initial symbiosis between robotics and microfluidic systems, each field has grown separately and independently, which could be attributed to the lack of a suitable interface between the macro-level (robotics) and the micro-level (lab-on-a-chip (LOC) devices)^43,44^. To date, only limited integration of robotics and microscale liquid manipulation methods exists, such as in digital microfluidics^42,45–47^, do-it-yourself (DIY) modified or commercially available inkjet printer heads, and robot-assisted electroporation devices to deliver a low volume of genetic materials^48^. More recently, a pipette-free microfluidic cap-to-dispense device connected to a robotic arm, which could dispense multiple droplets along user-defined trajectories was demonstrated^44^. In this article, we present a robot-assisted acoustofluidic end effector (RAEE) system combining both robotics and acoustofluidics functions. Our system comprises a robotic arm combined with an acoustofluidic device as an end effector. The new capillary-based acoustofluidic device, driven by ultrasound, creates oscillations within the capillary body and exhibits controlled flow profiles or microstreaming when the capillary is immersed in liquid. The acoustofluidic end effector yields two unique microstreaming profiles: (i) a helical vortex or a corkscrew-type fluid motion generated along the long axis of the capillary and (ii) and a frequency-dependent 3D microstreaming produced at the tip of the capillary. We showcased the RAEE’s multifunctional capabilities by executing liquid pumping, droplet merging, selective microparticle trapping, and mobile viscous mixing of complex viscous liquids. We further demonstrate the trapping of a zebrafish embryo by our acoustofluidic device. Finally, we pre-programmed the RAEE to perform automated mixing of viscous liquids in well plates, demonstrating that it can provide a powerful and versatile platform for achieving efficient, rapid, and automatic execution of the chemical processes typically desired in most modern laboratories.

## Results

### Working principles of robot-assisted acoustofluidic device

**Fig. 1:**
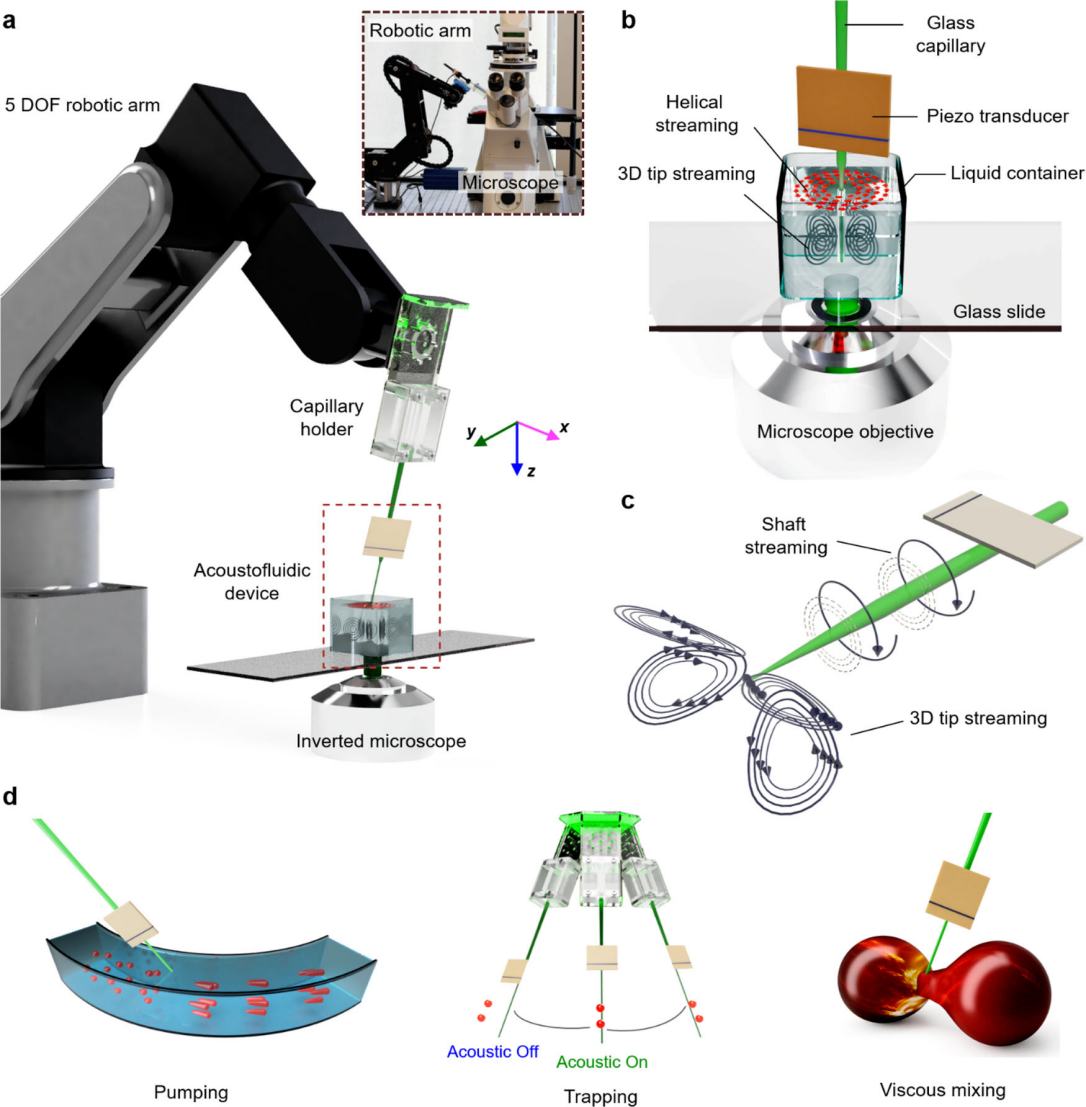
Experimental set-up and working principle of the robot-assisted acoustofluidic end effector (RAEE) device. **a** The RAEE device is comprised of a glass capillary and piezo-electronic transducer connected to a five-axis robotic arm. The whole set-up was mounted on an inverted microscope using a capillary holder. The inset illustrates the overall experimental set-up. **b** An enlarged view of the RAEE device when submerged in liquid with a focus on streaming profiles, especially the out-of-plane streaming. **c** The acoustofluidic device generated two distinct flow profiles: (i) circular-flow fields along the shaft of the capillary, and (ii) frequency-dependent 3D streaming at the tip of the glass capillary. **d** We have applied the RAEE to execute pumping, selective trapping, and viscous mixing.

Figure 1a shows our robot-assisted capillary-based acoustofluidic device. Our acoustofluidic device, i.e., the end effector comprised a hollow borosilicate capillary with an outer diameter of 1500 μm at the base, tapering to ~3–10 μm at the end (tip), coupled with a piezo electronic transducer (see also Supplementary Movie 1). The piezo transducer generated acoustic waves based on input from an electronic function generator that regulated frequency and amplitude, thereby controlling the oscillation of the capillary. The excitation frequency of the acoustic waves was modulated from 5 to 300 kHz while maintaining an applied peak-to-peak voltage of 1–20 *V*_PP_. The acoustofluidic device was connected to a custom-built 3D-printed holder via a syringe to a five-axis robotic arm. The entire set-up was positioned adjacent to an inverted microscope and the system was studied through fluorescent microscopy. The device was characterized by immersing it into a liquid chamber containing 2.0 and 5.0 μm tracer particles (Fig. 1b) and capturing the experimental results using light-sensitive and high-speed cameras.

### Helical acoustic streaming along the capillary

When the immersed acoustofluidic end effector was subjected to ultrasound, it produced asymmetric oscillations that created intricate vortices in liquid, a phenomenon often referred to as acoustic microstreaming. Two distinct steady-streaming profiles developed at different locations along the capillary: (i) helical streaming at the tapered region of the capillary shaft and (ii) frequency-dependent 3D streaming at the tip of the capillary (Fig. 1c). The streaming generated by the acoustofluidic device combined with the robotic arm allowed the execution of various applications, such as performing liquid pumping, selective particle trapping, and viscous mixing, as shown schematically in Fig. 1d.

**Fig. 2:**
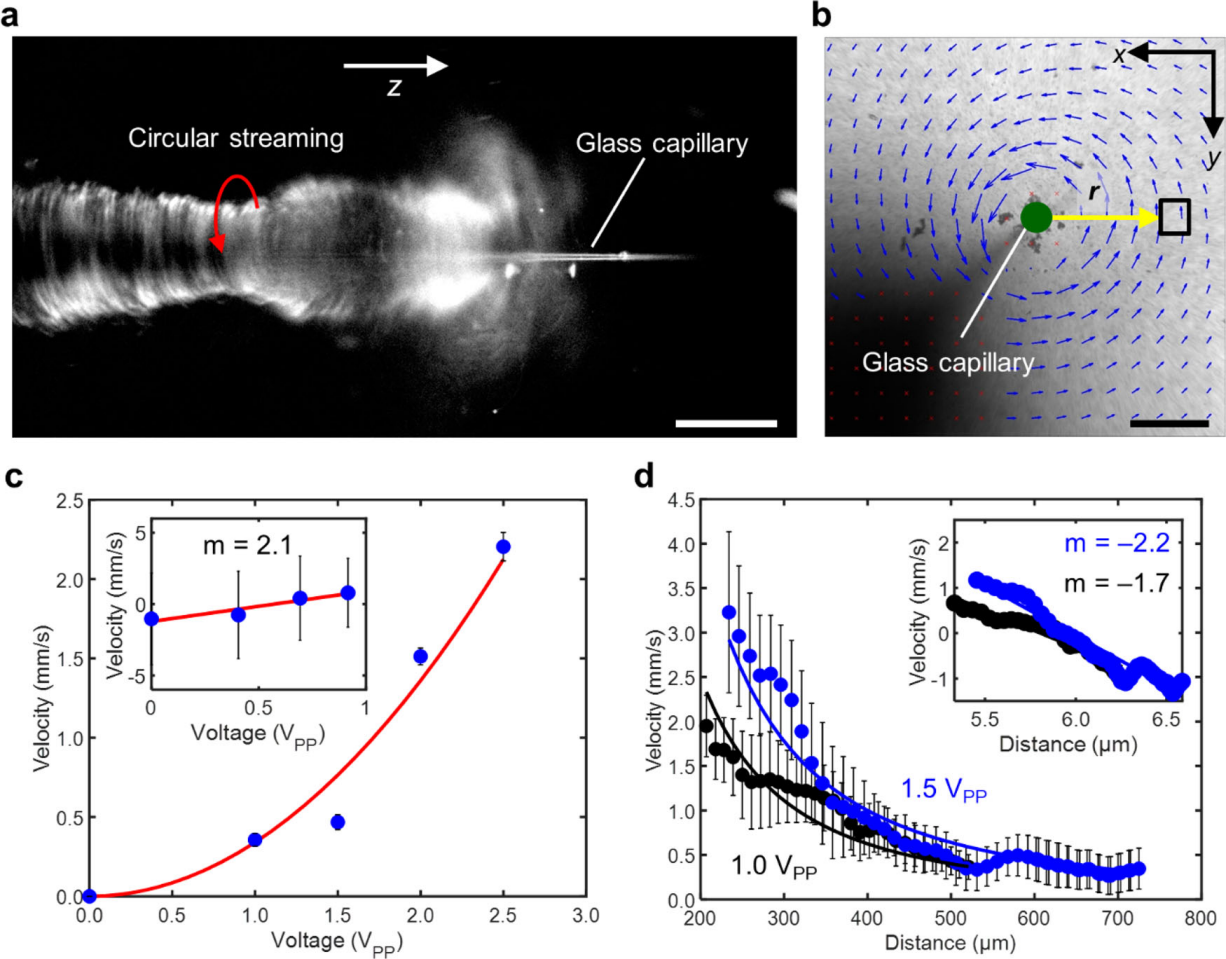
Characterization of the circular streaming in an acoustofluidic device. **a** Randomly distributed tracer microparticles were attracted towards the capillary and exhibit out-of-plane circular streaming along the shaft of the glass capillary in the *z*-axis. The red arrow depicts the direction of movement of microparticles. See Supplementary Movie 2. Scale bar: 30 µm. **b** The acoustofluidic device generated a circular-flow field in the liquid surrounding its body, indicated by the PIV-generated velocity fields. The PIV was generated using MATLAB code and the blue arrows represent the velocity and direction of the microparticles. The green dot represents the position of capillary tip. See Supplementary Movie 2. Scale bar: 500 μm. **c** Average velocities of the tracer particles at the site indicated by the square box in **b** versus the voltage applied. Particle velocities were proportional to the square of the voltage used, thus satisfying the quadratic relation, as demonstrated in the log plot (inset). **d** Average velocities of tracer particles versus distance from the capillary boundary. Velocities scale to the power of negative two with distance. Each data point represents the average velocity measured from at least eight tracer particles. Error bars in both graphs **c**, **d** represent standard deviation (s.d.) as *n* ≤ 5. See Supplementary Data Files 2 and 3 for the source data for both graphs.

In this section, we will describe the streaming developed along the shaft, i.e., the long axis of the capillary. When the submerged device was activated at an excitation frequency of 50 kHz and amplitude of 20 *V*_PP_, its oscillations attracted the tracer particles, which then followed the trajectories of the flow field, executing an out-of-plane vortex around the capillary (as depicted in Fig. 2a). A closer inspection reveals that the tracer particles not only exhibit a circular motion but also execute a spiral motion along the long axis of the capillary; this effect is imaged from in Fig. 2b, c (see also Supplementary Movies 2 and 3). The spiralling velocity of the tracers rapidly increases as the particles move forwards in the narrower tip region of the glass capillary, likely due to larger oscillations of the narrower tip region. The capillary device was then rotated by 90° to visualize the overall streaming flow profile (Fig. 2a). Figure 2b shows the velocity field of the capillary as viewed from the *x*–*y* plane (top view), confirming the circular flow field exhibited by the tracer particles. The Reynolds number of our acoustofluidic device was estimated as ${Re}={uD}/{{{\rm{\nu }}}}\,\approx \, 0.07$, where $u\,\approx \,2.0{{{\rm{mm}}}}/{{{\rm{s}}}}$ is the streaming velocity, $D\,\approx \,2.07\,\upmu {{{\rm{m}}}}$ is the diameter of the tracer particles, and ${{{\rm{\nu }}}}\,\approx\, {10}^{-6}\,{{{{\rm{m}}}}}^{2}/{{{\rm{s}}}}$ is the kinematic viscosity of water at room temperature. This value suggests that the system is viscous-dominated, which was further confirmed by the tracers coming to a halt instantaneously when the acoustic wavefield was turned off, suggesting a lack of inertia.

The strength of the circular flow field produced by the capillary is determined by the intensity of the ultrasound, which we demonstrated by adjusting the voltage applied to the piezo transducer (as seen in Fig. 2c). The acoustic streaming is a time-averaged (second-order) response of the fluid systems that is driven by force and mass sources arising out of (first-order) acoustic response of the fluid. The driving force and mass sources depend quadratically on the first-order pressure and velocity, which in turn depend linearly on the applied voltage^49^. Consequently, the streaming velocity, *u* is expected to scale quadratically with applied voltage, *V*_PP_ (i.e. $\propto { \sim V}_{{{{\rm{PP}}}}}^{\,2}$). We characterized tracer velocity as a function of acoustic power at a distance of 500 μm from the centre of the capillary, indicated by the black box in Fig. 2b. The velocities of tracer particles were measured using a custom-built PIVlab MATLAB script. Figure 2c shows that the tracer particles circulate with velocities in the range of a few mm/s, and the plot suggests that the expected quadratic relation is reasonably well-satisfied by the acoustofluidic device. Next, we investigated the scaling of streaming velocity with distance from the centre of the glass capillary. Figure 2d shows scaling of streaming velocity versus radial position at 1.0 and 1.5 *V*_PP_, indicated by black and blue dots, respectively. When close to the tip, the angular velocities are largest and scale with the inverse square of the distance from the tip (i.e. streaming velocity, $\propto \sim 1/{r}^{2}$). The relatively large standard deviation in each data point in Fig. 2d can be attributed to the difficulty in estimating tracer velocities close to the tip, which arises from the poor fluorescent sensitivity of the high-speed camera. We observed a counter-clockwise circular flow field for most acoustic excitation frequencies, which could be attributed to the piezo transducer being bonded on one side of the capillary.

### Frequency-dependent streaming at the capillary tip

Numerous objects produce nonlinear acoustic streaming in the surrounding liquid environment when exposed to ultrasound; examples include gas-filled microbubbles^34,35,50–52^ and solid structures^17,49^. For both microbubbles and solid objects, the precise nature of the oscillation (e.g., lateral, radial, or a combination thereof) is crucial in determining the ensuing flow patterns. While numerous bubble-based streaming systems have been reported previously for a variety of microfluidic applications, harnessing the oscillations of a solid object remains an attractive alternative due to the inherently unstable nature of bubble-based systems. Here, we subjected a capillary to ultrasound to develop frequency-specific 3D streaming flow profiles at its tip.

**Fig. 3:**
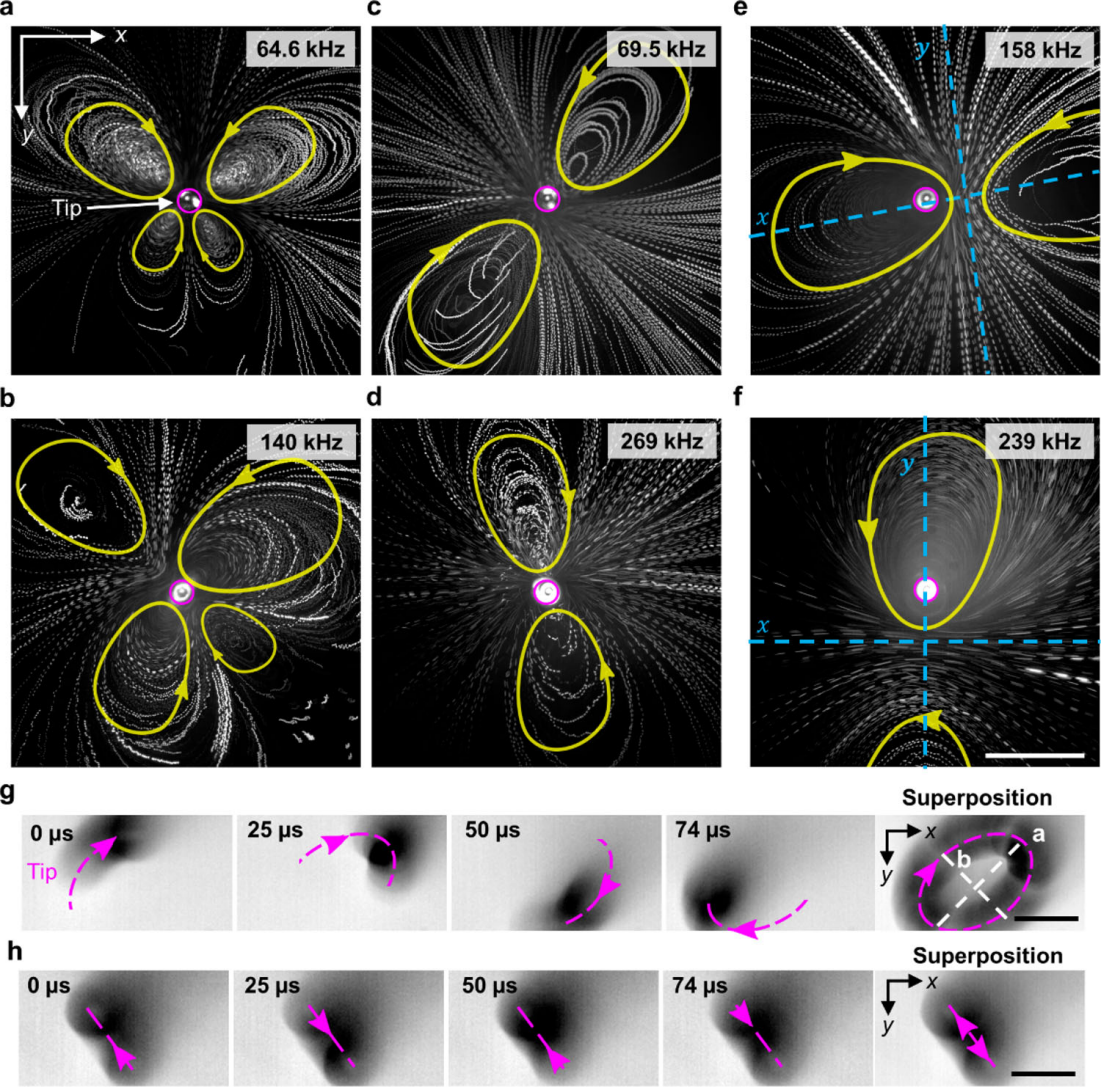
Frequency-specific vortices or streaming produced by the tip of the acoustofluidic device in water containing tracer particles. The acoustofluidic device produced steady 3D vortex flow patterns comprised of counter-rotating vortices (The pink circle represents the capillary tip position and yellow geometry represents the direction and path of motion of particles.) (see Supplementary Movie 4): a butterfly-like pattern of four vortices at **a** 64.6 kHz and **b** 158 kHz; a pair symmetric about the tip at **c** 69.5 and **d** 269 kHz; and a pair ~100 µm off-centre from the tip at **e** 69.5 kHz and **f** 269 kHz. The direction of flow is marked with yellow arrows, and the capillary tip indicated by a magenta circle. The symmetry axis is indicated in blue for images where the capillary tip does not represent the centre of symmetry. Dotted magenta lines indicate tip oscillation of the acoustofluidic device (see Supplementary Movie 5), which undergoes **g** elliptical motion at excitation frequency 6.8 kHz and amplitude 20 *V*_PP_ and **h** translational motion at 7.8 kHz and 20 *V*_PP_. Scale bars: 50 µm.

Referring to Fig. 3, the frequency-dependent 3D streaming profiles observed at the capillary tip. These profiles comprise a varying number of clockwise and counter-clockwise streaming patterns, as shown in Fig. 3 (see also Supplementary Movie 4). Figure 3a, b demonstrate the intricate, butterfly-like vortex flow patterns consisting of four counter-rotating vortices that resulted when the capillary was exposed to ultrasound at frequencies of 64.6 and 140 kHz, respectively. The magenta circle indicates the capillary tip, while the yellow arrows mark flow directions. In contrast, excitation frequencies of 69.5 and 269 kHz, depicted in Fig. 3c and d respectively, yielded only a pair of counter-rotating vortices that were symmetric about the tip. Although these two streaming profiles are similar, the one in Fig. 3d is transformed rotationally by 135° relative to that in Fig. 3c. Interestingly, while most of the developed flow patterns were symmetrical around the tip, some exceptions were also observed. For example, Fig. 3e, f demonstrate the streaming flow profiles that resulted at excitation frequencies of 158 and 239 kHz, respectively. These counter-rotating vortices are still symmetric, but not about the tip; that is, the tip position is ~100 µm off-centre in the direction of the left vortex (in Fig. 3e) or top vortex (in Fig. 3f), the second profile being rotationally transformed by ~90° relative to the first. The strength (i.e. the velocity and size) of the streaming profile was observed to scale with the applied power, but the streaming pattern or signature remained consistent for a given frequency.

We hypothesized that these varying vortex flow patterns arise due to the capillary tip oscillating in different patterns. Since direct imaging of those oscillations is challenging when applying high-frequency ultrasound, we employed lower frequencies and used a high-speed imaging camera to capture the oscillation of a capillary tip in air. We, therefore, investigated the oscillation of the capillary from the front and side. When a capillary was exposed to ultrasound in air, a standing wave was observed with a noticeable oscillation amplitude at the free capillary tip. Figure 3g, h show two representative oscillation patterns where the tip was observed to oscillate in an elliptical (*f* = 6.8 kHz) or translational pattern (*f* = 7.8 kHz), respectively (Supplementary Movie 5). These results point to the oscillation profile of the capillary tip being frequency-dependent, which further results in frequency-dependent streaming flow patterns (see also Supplementary Note 1 and 2 and Supplementary Figs. 1 and 2). Overall, these observations highlight the ability of the RAEE to generate controlled streaming patterns of varying strengths near the capillary tip, affected by modulating the actuation frequency and acoustic power (see also Supplementary Fig. 3).

### Robot-assisted acoustofluidic manipulation system

**Fig. 4:**
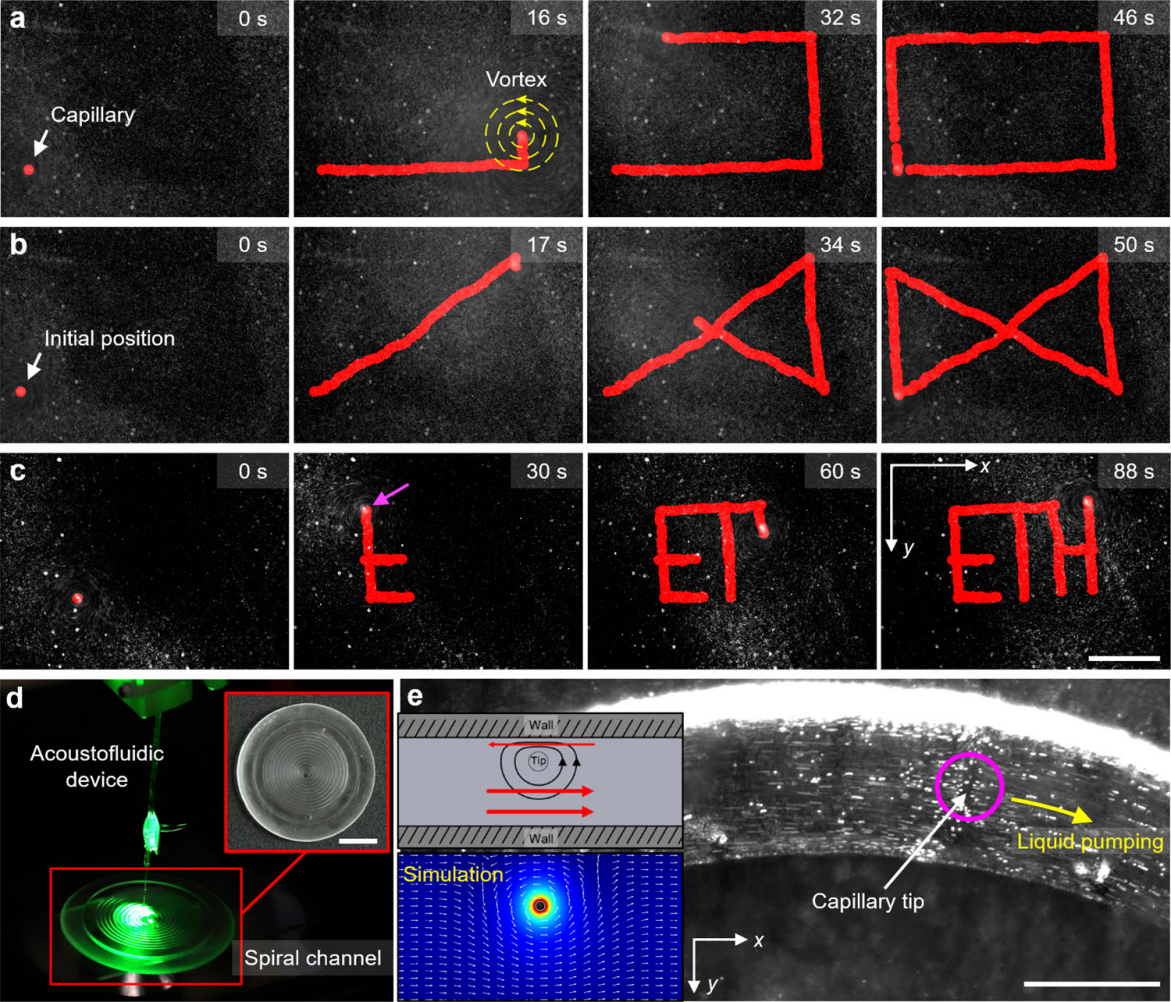
Arbitrary controlled motion of the mobile microvortex generator and design of the liquid pump. Image sequences depicting the robot-assisted acoustofluidic device executing automated patterns (here the red marking represents the path of the capillary tip and yellow concentric circles represents the vortex generated) (see Supplementary Movie 6): **a** a rectangle, **b** an hourglass, and **c** letters creating the acronym “ETH.” **d** Photograph showing the RAEE-based microfluidic liquid pump, comprised of an acoustofluidic device and a 3D-printed PDMS-based fluidic channel (see Supplementary Movie 10). **d** Transformation of the mobile microvortex into a LOC micropump upon immersion of the acoustofluidic device into a 3D-printed spiral fluidic channel. **e** The vortices produced at an excitation frequency of 134 kHz and amplitude of 10 *V*_PP_ generated liquid pumping in the left-to-right direction (the magenta circle represents the capillary tip position and yellow arrow represents the liquid pumping direction) (see Supplementary Movie 10). The top inset demonstrates the pumping mechanism (here the red arrow represents the direction of particle motion in that region). The bottom inset illustrates the COMSOL simulation of fluid pumping when the capillary tip was introduced adjacent to the wall. Scale bar **a**–**c**: 400 µm, scale bar **d**: 10 mm, scale bar **e**: 200 µm.

Combining the on-demand controlled vortex generation of the acoustofluidic device with a robotic arm for discretionary positioning promises to open up exciting novel possibilities for a 3D mobile platform with applications in mobile micropumps, micromixers, selective particle trapping, and droplet merging. We first demonstrated a mobile micro vortex generator of the acoustofluidic end effector and investigated its control capabilities. The robot-assisted acoustofluidic device can be manipulated to trace an arbitrary, user-defined shape in both 2D and 3D space. We programmed the robotic arm’s control unit with automated patterns such as a rectangle (Fig. 4a and Supplementary Movie 6), an hourglass (Fig. 4b and Supplementary Movie 6), and moved along a path to write “ETH” (Fig. 4c and Supplementary Movie 6). The positioning of the robotic arm utilized inverse kinematics. The speed and targeted end-effector positions were controlled using trajectory control, with the trajectory tracked as red dots. The initial positions, i.e., the capillary and the corresponding vortex, were indicated by a red dot in the first frame of each image sequence. To ensure the precision and accuracy of movements, the robotic arm was manipulated at a low velocity of 1 mm/s; and to ensure continuity of motion through sharp corners, a delay of 1 s was programmed before executing a turn. The flow pattern can further be manipulated in a 3D space with excellent accuracy and precision, contributing to fast and reliable manipulation. Ultimately, the position and velocity of the mobile micro vortex generator can be manipulated by controlling the robotic arm, and the strength of the micro vortex; the latter is determined by the voltage applied to the transducer connected to the acoustofluidic device (see Fig. 2c).

### Liquid pumping

Pumping of viscous liquids at the micro- to nanoscale is essential in sample processing, bioanalysis, diagnostic, and therapeutic applications. To date, several microfluidic pumping mechanisms have been developed that employ surface tension^53^, electric fields^54^, magnetic fields^55^, optical tweezing^56^, thermal effects^57^, periodic volume displacement^58^, and acoustics^5^. Nonetheless, despite the advances in this field, many challenges still remain, including achieving particle independence, microfluidic channel deformation, and high-throughput pumps for viscous fluids^59–64^. Acoustic fluid pumping methods based on breaking the symmetry of acoustic streaming patterns have solved some of these issues, but such approaches require fabrication of special structures inside the microchannels (e.g., sharp-edged structures) and therefore difficult to achieve liquid pumping in arbitrary microchannels. The current method circumvents the need for such specialized fabrications and so can perform pumping within any microfluidic enclosure, as demonstrated for a spiral channel in Fig. 4d. Furthermore, the fluid pumping rate can be easily controlled by adjusting the applied voltage to control tip oscillation. This capacity for operational control, together with precise robotic control of tip position, positions our micro-acoustofluidic end effector to potentially serve as an easy and versatile technique for achieving pumping in routine microfluidic workflows.

We transformed our RAEE-based micro vortex generator into a micropump by immersing the acoustofluidic end effector into a 3D-printed spiral fluidic channel (see Fig. 4d). In general, the acoustic energy from tip oscillations dissipates both in the bulk fluid and in the boundary layer regions adjacent to the oscillating surface within the fluid domain. Based on the high frequency of oscillations (resulting in large wavelengths compared to the tip length scale) in the current work, we believe that acoustic streaming is primarily driven by boundary layer dissipation (see also Supplementary Note 2)^39^. The boundary-layer dissipations then cause outer streaming flows as shown in Fig. 4. In the case of pumping fluids, we break the symmetry of the problem by employing the interactions between the acoustic streaming vortices and the channel walls. We position the oscillating tip closer to one side of the wall to achieve liquid pumping, as shown in Fig. 4e. As a result, the circular streaming pattern around the tip produces a net unidirectional liquid motion. Thus, when the acoustically-activated capillary (excitation frequency 134 kHz, amplitude 10 *V*_PP_) was brought close to the channel’s wall by the robotic arm, the vortex symmetry normally present in the helical streaming around the capillary was broken. As a result, the RAEE initiated a net liquid flow or pumping in the order of mm/s, illustrated by the 6 μm tracer particles in Fig. 4e (see also Supplementary Movie 10). Pumping occurred from left to right, which is attributed to the acoustofluidic end effector producing CCW streaming and the capillary being brought towards the top sidewall of the channel. Furthermore, the flow rate can be actively controlled by altering the voltage applied to the piezo transducer, which scales quadratically with the applied voltage. As the flow was viscous in nature, the pumping ceased immediately when the acoustic field was turned off. In light of the precise robotic control of the acoustofluidic end effector, RAEE represents an affordable and versatile method for liquid pumping without requiring any specialized fabrication^65,66^.

### Selective trapping of microparticles

Trapping and transport of microparticles and cells has significant applications in biological micromanipulation, cell assays, and cell–cell interactions^15^. Several acoustic-based trapping mechanisms have been developed to trap and manipulate microparticles, such as using surface^31,67^ and bulk^68^ standing acoustic wavefields to trap microparticles in their pressure node arrays. However, achieving selective and dynamic manipulation has always been a challenge. An acoustic trapping device capable of picking and moving single cells was developed by depositing spiral electrodes on a piezoelectric substrate; however, this method has no specificity in terms of what particles it traps. Methods using acoustically activated microbubbles and sharp-edged structures have also been demonstrated to trap microparticles, and an array of acoustically activated gas-filled microbubbles has been used to manipulate microparticles. However, these systems suffered from the instability of microbubbles, are harmful to living cells, and offer only limited manipulation capability as they are not easily translated to settings other than microfluidic chambers. Here, we demonstrate a capillary-based acoustofluidic system with the new functionality of selectively trapping microparticles based on their size. Furthermore, since the system is attached to a robotic arm, we can programme the device to follow any trajectory in 3D.

**Fig. 5:**
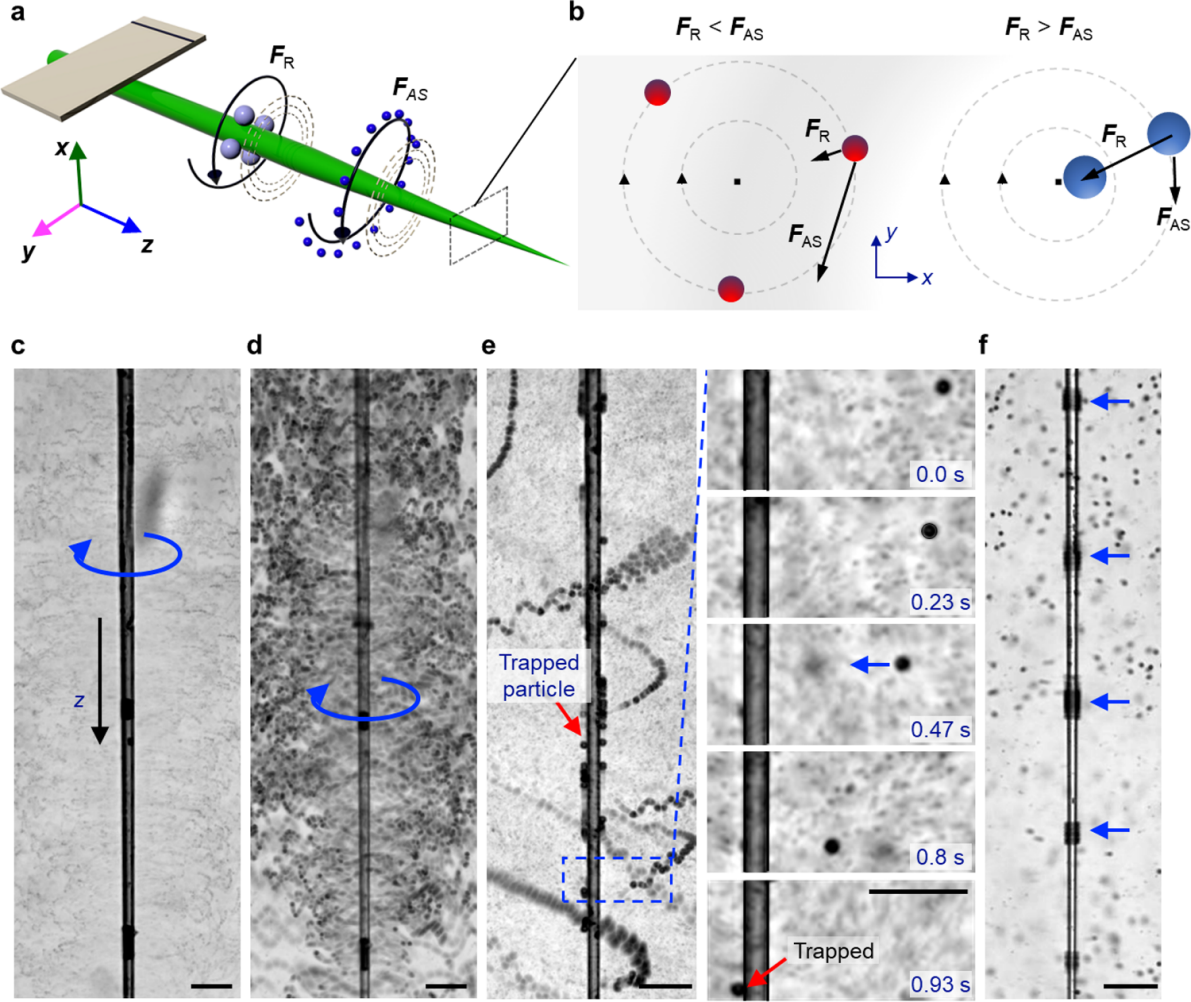
Interaction and selective trapping of microparticles in an acoustically activated glass capillary. **a** Schematic illustrating the selective trapping of microparticles along the acoustofluidic capillary system. Large particles, illustrated in red, relocate and get trapped along the shaft of the glass capillary due to the radiation force (**F**_R_). Small particles, shown in blue, follow out-of-plane circular streaming (**F**_AS_) around the capillary shaft. **b** (Left panel) The acoustic streaming force dominates for particles with diameter ${a} \, < \,{a}_{c}$. (Right panel) The radiation force dominates for particles with diameter $a \, > \,{a}_{c}$. (blue arrow represents the direction of the movement of microparticles) **c**, **d** Image sequence demonstrating circular streaming of 2 and 10 µm polystyrene microparticles around a glass capillary at an excitation voltage of 100 kHz and amplitude 3 *V*_PP_ (blue arrow represents the direction of the movement of microparticles and red arrow shows the trapped particles) (see Supplementary Movie 7). **e** Superimposed time-lapse images illustrating trapping of polystyrene microparticles along the shaft of a glass capillary at 120 kHz and 10 *V*_PP_. The image series zooms in on the area marked with a blue box and shows the trapping of a single 15 µm microparticle. **f** Trapping of 10 µm beads at the pressure nodes of a glass capillary at an excitation frequency of 270 kHz and amplitude 10 *V*_PP_ (see Supplementary Movie 7) (here the blue arrow shows the location of particles). All scale bars: 100 µm.

Microparticles such as polystyrene, cells, organisms, and small animal models placed near an acoustically activated glass capillary experience predominantly acoustic radiation force (F_R_) and acoustic streaming-induced drag (F_AS_), as shown in Fig. 5a. The scattering of incident acoustic waves by the oscillatory capillary causes an acoustic radiation force to act on particles. While the exact acoustic radiation force field depends on a variety of factors, including the geometry and dimensions of the capillary and its vibration profile, the acoustic radiation force on the particle can be expected to scale with the volume of the particle, as suggested by prior reports^39^ (see also Supplementary Note 3). In addition, viscous attenuation of the scattered capillary-generated acoustic waves results in acoustic streaming in the surrounding liquid. The streaming-induced drag force can be scaled as the Stokes’ drag since the Reynolds number (${Re}={av}\rho /\mu=0.01,$ where *a* and *v* are the particle’s diameter and velocity, respectively, and *ρ* and *μ* are the liquid’s density and dynamic viscosity, respectively) is small due to the small dimensions of the microparticle. As a result, the acoustic streaming force scales with particle diameter. By taking the ratio of the two forces, we can predict what sizes of particles will migrate to the surface of the capillary. For example, streaming dominates when the ratio of acoustic radiation force to streaming is less than 1, i.e., **F**_R_/**F**_AS_ < 1, and the particles do not become trapped but rather undergo helical streaming (Fig. 5b, left panel). And, the radiation force dominates when the ratio of acoustic radiation force to streaming is greater than 1, i.e., **F**_R_/**F**_AS_ > 1, and the particles are trapped (Fig. 5b, right panel).

We studied the particle–capillary interaction by injecting different-sized polystyrene microparticles into the liquid bath containing the capillary. When a solution containing 2- and 10-µm polystyrene microparticles was dispersed in the liquid bath and the capillary activated at 120 KHz and 10 *V*_PP_, the microparticles underwent helical streaming (Fig. 5c, d and Supplementary Movie 7). When we injected microparticles with a diameter of 15 µm, those larger microparticles did not execute continuous helical streaming in response to acoustic activation. Instead, the microparticles accelerated towards the wall and became trapped at the capillary wall within seconds, as illustrated in the image sequence of Fig. 5e (see also Supplementary Movie 7). Shown in Fig. 5e is the trapping of a high concentration of 15-µm polystyrene particles, which are distributed unevenly over the surface of the capillary. We found our analysis to be consistent with our experimental results, i.e., the 2- and 10-µm polystyrene microparticles executed out-of-plane streaming, while the 15-µm particles were trapped. Since there is no analytical solution for the radiation force of a vibrating capillary, it is difficult to determine the cut-off size above which microparticles become trapped. The minimum diameter of a particle that can be trapped can be estimated by setting **F**_R_ = **F**_AS_.

Interestingly, when we increased the voltage applied to the piezotransducer, i.e., the amplitude of the ultrasound, we observed smaller 10-µm microparticles to become arranged in bands almost equidistantly spaced along the capillary, Fig. 5f. Interestingly, these microparticles are not stationary, but execute circular motion around the circumference of the capillary at fixed locations. The microparticles were grouped in regions where the vibrational amplitude was at minimum, i.e., pressure nodes. The separation between each band was measured to be $\sim 270\pm 42$ µm. These trapping points thus further suggest that a standing wavefield is developed along the capillary. Such a wavefield becomes prominent when the capillary is acoustically activated in air; see also Supplementary Movie 8.

### Trapping of zebrafish embryo

**Fig. 6:**
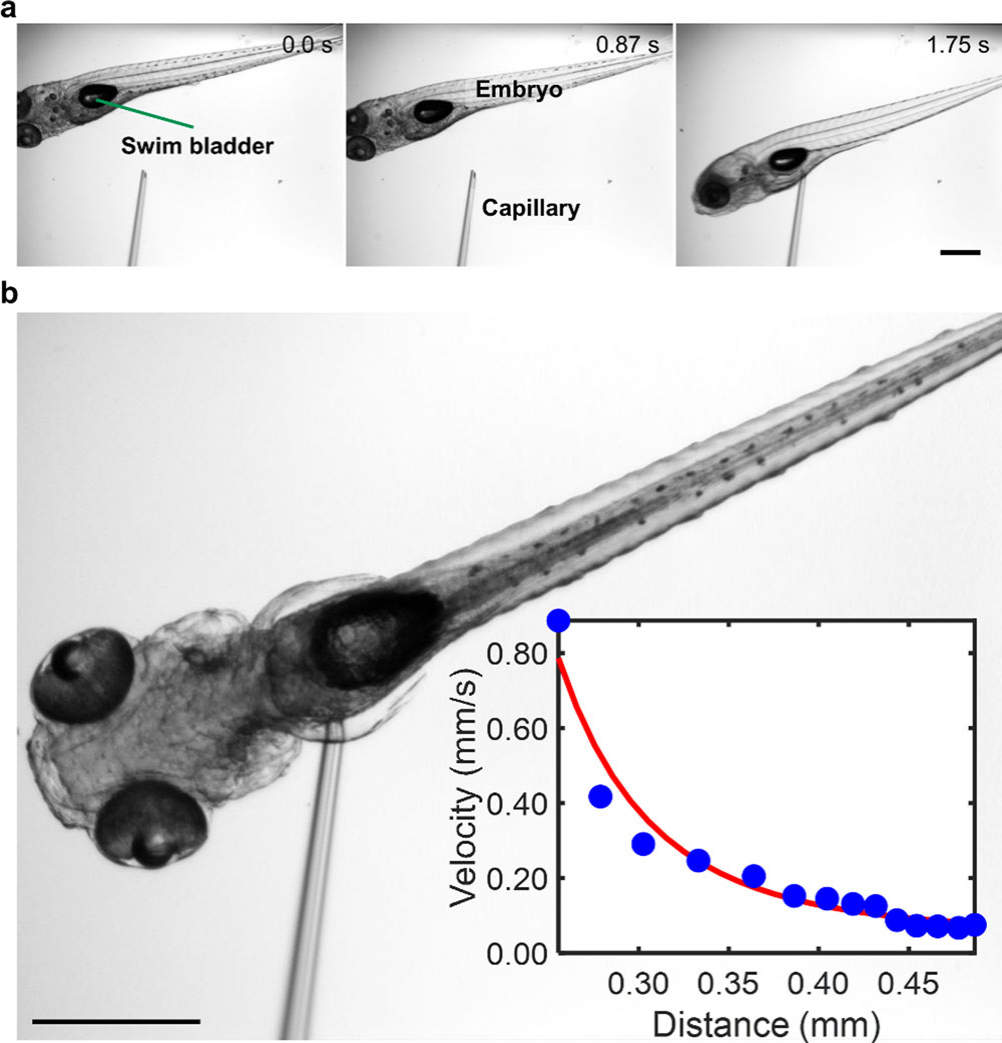
Trapping of a zebrafish embryo using the RAEE. **a** Image sequences demonstrating the trapping of a zebrafish embryo (120 hpf) containing an air-filled swim bladder by the acoustofluidic end effector, activated at an excitation frequency of 80 kHz and voltage of 20 *V*_PP_ (see Supplementary Movie 9) (the green arrow depicts the swim bladder). Scale bar: 200 µm **b** A magnified micrograph of a trapped zebrafish embryo. Inset: plot of embryo translation velocity versus distance from the capillary tip. Error bar represents that the standard deviation (s.d.) *n* = 14. See Supplementary Data File 4 for source data for the graph. Scale bar 200 µm.

Precise and controlled trapping and manipulation of mm-sized model organisms such as *C. elegans*, zebrafish, and Drosophila embryos is a challenging but essential requirement for applications in high-throughput drug screening^69,70^, imaging^71,72^, and sorting^73,74^. Zebrafish embryos are excellent substitutes for higher mammalian models. They are used frequently to understand the molecular mechanisms involved in human diseases and identify cures widely used for pharmaceutical purposes^75^. However, routine positioning of zebrafish embryos currently requires a skilled operator and is very tedious, involving multistep manual manipulation using micropipettes on agarose gel. Moreover, the embryos are often damaged by this technique because uncontrolled handling exposes them to excessive stress^8,75^. Accordingly, recent advances in trapping zebrafish embryos have generated a great deal of interest. These include vacuum, hydrodynamic, and capillary-suction methods, gravity-induced docking on microfluidic wells, electrowetting on a dielectric-based device, and applications of ultrasound acoustics^2,8,75^. Ultrasound in particular has recently emerged as an exciting strategy for trapping zebrafish embryos due to its safe operation at moderate levels of acoustic pressure, non-invasiveness, ability to generate large trapping forces on the order of micronewtons, ease of miniaturization, and low cost of fabrication For example, Chen et al. developed an interdigitated surface acoustic wave device to trap a zebrafish embryo using a vortex^8^. Also, an acoustic levitation device has been developed to trap a zebrafish embryo (5 hpf) in mid-air for fluorescent light sheet microscopy^8^. Here, we have designed a trapping device based on the acoustic radiation force emitted from an oscillating glass capillary. The integration of this device with a robotic arm allows us to trap and manipulate an embryo according to any pre-programmed trajectory in 2D and potentially in 3D. We demonstrate the RAEE’s versatile nature by trapping an anaesthetized 120 h post-fertilization (hpf) zebrafish embryo. The acoustofluidic end effector was brought near the embryo and activated when the distance between the capillary tip and the fish was approximately mm. We observed the embryo to be strongly pulled to the acoustofluidic device, i.e., it became trapped at the capillary tip, as shown in the image sequence in Fig. 6a. Figure 6b shows a magnified micrograph of a trapped zebrafish, highlighting the specific attraction of the swim bladder to the capillary tip (see also Supplementary Movie 9). The inset depicts the rapid increase of the embryo’s translational velocity as it approaches the capillary tip; specifically, the embryo’s speed varies with the fourth power of distance. Terminating the applied acoustics releases the trapped embryo from the tip without visible harm. Embryos lacking swim bladders are also attracted to the capillary tip (see also Supplementary Movie 9). As discussed earlier, when a solid microparticle is near an acoustically activated object such as a microbubble, the particle experiences both radiation and streaming forces; the radiation force scales with the volume of the particle, while the streaming force scales with its diameter^76^. Since the size of the zebrafish embryo is on the order of millimetres, the radiation force is expected to dominate and, responsible for trapping. We can selectively trap and manipulate embryos according to their size, shape, and age by incorporating a microscopic visual feedback system. Thus, our method supports capturing zebrafish in an automated fashion, reducing the need for manual handling, and the robotics also allow better manoeuvrability.

### Pre-programmed high-throughput mixing of viscous liquids

The development of mixing technologies that can achieve uniform and rapid mixing of viscous liquids at the microscale will open new avenues in food processing, the pharmaceutical industry, and additive manufacturing^1^. Although recent reports describe mixing viscous liquids at microscales^1^, most existing micromixers have limitations in handling viscous liquids. Injecting liquid is already a challenging task at the microscale; mixing viscoelastic liquids remains an even greater challenge because of the lack of inertia associated with extremely low Reynolds numbers. As a result, there exist very limited micromixers that can mix viscous fluids and to date most existing systems utilizes acoustics. The acoustic microstreaming of microbubbles in microfluidic devices, for example, was used to mix DI water with a glycerol solution^77^ and DI water with PEG-700^78^. A micromixer has been developed that leverages bubble inception and inertial cavitation from the surface roughness of a channel’s sidewall to mix two viscous liquids. An acoustic microstreaming method that uses oscillations generated by sharp edges built into the microfluidic devices was utilized to mix water and sputum, a biospecimen that is 10 times more viscous than water^79^. In addition, active microscale mixing is achieved by rotating an impeller in the nozzle of a 3D printer to mix multiple inks while 3D printing^1,80^. Current micromixers are constrained by unstable microbubble, random siting of microbubble initiation; furthermore, mixing is developed in enclosed systems, thus, greatly limiting its application in life sciences that require operation in open systems, such as well-plates.

Our developed micromixer is unique in that it has two distinct regions for mixing: (i) single or multiple frequency-specific counter-rotating vortices at the capillary tip, and (ii) streaming developed around the capillary shaft. The streaming at the tip can be used for mixing droplets and mixing in open microfluidic devices, while the combination of tip streaming and eddies around the shaft promotes the mixing of viscous fluids in 3D deeper microwells. Additionally, as the capillary-based acoustofluidic device is connected to a robotic arm, we are able to programme arbitrary mixing paths in order to achieve a high level of micromixing and automate high-throughput mixing processes.

**Fig. 7:**
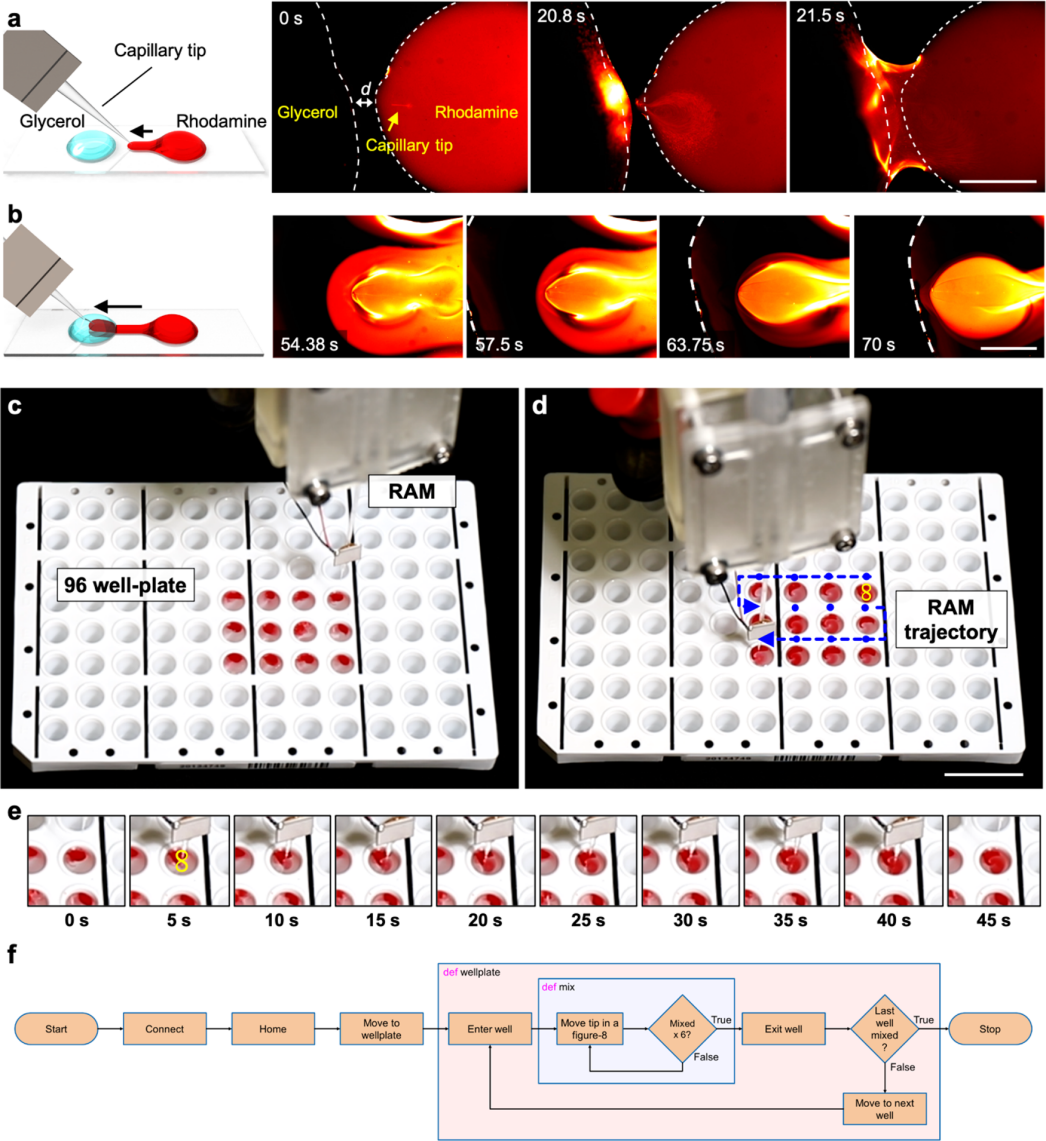
Pre-programmed high-throughput mixing of viscous fluids in a 96-well plate using the RAEE. **a** Conceptual schematic showing the merging of two droplets (left). Image sequences demonstrate the merging of glycerol (black) and rhodamine solution (red) droplets. Dotted white lines indicate droplet contours. Scale bar: 500 µm **b** Schematic showing the mobile mixing of rhodamine and glycerol droplets. MAEE was utilized to achieve mobile mixing of rhodamine solution in a glycerol droplet. Dotted white line indicates the contour of the glycerol droplet. **c** The RAEE system in the initial position above the plate, prior to mixing. **d** Device trajectory for the mixing process, indicated by blue dots and arrows. Movement that occurs while mixing inside a well is indicated by yellow lines (see Supplementary Movie 13). Scale bar: 20 mm. **e** Mixing procedure in a representative well, where the frame at 0 s indicates the unmixed state and that at 45 s indicates the mixed state. The yellow line indicates the path of the RAEE system inside the well while mixing (see Supplementary Movie 13). **f** The schematic illustrates the algorithm used by RAEE to achieve homogenous mixing in a 96-well plate.

Here, we demonstrate an active, mobile method for mixing viscous liquids. Figure 7a shows a pair of droplets, one containing rhodamine solution (right) and the other glycerol (left), separated by ~100 μm apart on a glass slide. The acoustofluidic device was initially positioned on the rhodamine droplet. Notably, glycerol has a viscosity that is six orders of magnitude larger than water (6.48 vs. 1.004 × 10^−^^6^ m^2^/s). The interface between glycerol and rhodamine droplets remains almost intact even after hours, indicating negligible diffusion-based mixing (see also Supplementary Fig. 4). The acoustofluidic device was initially positioned on the rhodamine droplet. As the robotic arm translated from right-to-left (from the rhodamine to the glycerol droplet) with the piezo transducer turned on, the rhodamine solution became trapped within the tip vortices and subsequently transferred into the glycerol droplet, upon which the two droplets merged and initiated vigorous mixing. As the capillary translated further right-to-left through the glycerol droplet, a halo-like mixed region of rhodamine solution in glycerol was produced (Fig. 7b, see also Supplementary Movies 11 and 12). The boundary between the mixed and unmixed parts was distinct, indicating a sharp interface, i.e. between mixed red and unmixed black. We executed active mixing at an excitation frequency of 44.9 kHz and amplitude of 20 *V*_PP_, which produced uniform mixing in milliseconds (see also Supplementary Fig. 4). All told, when mounted on a robotic arm, our acoustofluidic device enables the mixing of viscous liquids through arbitrary and predefined paths, and potentially the local mixing of liquids in a 3D space.

A 96-well plate was filled with glycerol and placed next to the robotic arm, aligned such that the coordinate axes of the arm correlated with the long and short sides of the plate. The robotic arm was equipped with the acoustofluidic device, which was in turn connected to a function generator and initialized. After all joints were homed, the robot was programmed to approach the 96-well plate. The acoustofluidic device was placed 10 mm above the centre of the first to-be-mixed well, as seen in Fig. 7c. Glycerol was dispensed into the wells of a 96-well plate, followed by the careful addition of 20 µl rhodamine solution to prevent any immediate mixing of the two substances. Subsequently, the piezo transducer was turned on and the robotic arm lowered the acoustofluidic device into the first well, then proceeded to move it in a “figure-8” pattern (each loop being 1 mm in diameter), as illustrated in Fig. 7d. The motion and the applied acoustics stirred the surrounding liquid, resulting in efficient mixing of the viscous fluids (Fig. 7e). After 40 s, the motion was terminated and the acoustofluidic device lifted to 10 mm above the well. The robot then proceeded to relocate the device above the next to-be-mixed well, where the procedure was repeated. Once all wells were mixed, the robot moved the device back to the initial position and terminated the mixing procedure (Supplementary Movie 13). Figure 7f illustrates the algorithm used by RAEE to achieve homogenous mixing. Because the vibration of the acoustofluidic device increases towards its tip, the best stirring occurs at the bottom of the well instead of the surface. This prevents a solid-body rotation of the viscous liquid and ensures mixing. While the mixing of samples in well-plates is a routine procedure in laboratories, common vortex mixer cannot handle viscous fluids. Our device can be integrated in a laboratory workflow as a viscous mixing station.

## Discussion

This article introduces the first acoustofluidic end effector for a robotic arm, thereby enacting an interface between macroscale robotics and microscale liquid and particle manipulation technology. The RAEE is a versatile platform that can find utility in a wide range of applications in chemistry, biology, and the life sciences. This new capillary-based acoustofluidic device yields two distinct steady-vortex flow profiles: (i) helical streaming or corkscrew-like motion along the capillary and (ii) frequency-dependent 3D streaming at the capillary tip. To demonstrate the system’s versatility and multifunctionality, we carried out pumping, droplet merging, selective microparticle trapping, and mobile mixing of viscous liquids. We further demonstrated trapping of zebrafish embryos. Finally, we preprogrammed the RAEE to perform automated mixing of viscous liquids in well plates, illustrating its versatility for the automatic execution of chemical processes.

The present work aimed at combining acoustofluidic physical principles with macroscale robotics to demonstrate a range of common laboratory functionalities; specifically, it exploits nonlinear acoustic streaming phenomena to achieve controllable fluid motion patterns. The frequency-dependent streaming flow profiles are produced from the oscillations of a glass capillary attached to a robotic arm, and this implementation is conceptually attractive on account of capillary oscillations being extremely stable and repeatable. Future work will provide a detailed investigation of the 3D nature of the produced flow profiles at the tip. We also aim in future to develop comprehensive numerical models pertaining to oscillation modes and corresponding streaming profiles.

We will further investigate 3D microparticle manipulation using our developed technology. Manipulating microparticles in three dimensions poses a fundamental challenge^15,81^. We already showed microparticles can be selectively trapped using capillary-based acoustofluidic device, and since it is attached to a robotic arm, it is possible to programme the device to follow any trajectory, i.e., to manipulate microparticles arbitrarily. Using such a method to precisely position cells in a 3D architecture will allow for novel functions in tissue engineering^82^. We also plan to use our technology to induce poration, which can lead to selective transfection through time-varying stress generated by acoustic streaming with high-throughput capability. And, we will also investigate the compound streaming profiles that result when using multiple capillaries. Since each capillary is paired with its own piezo transducer, programmable streaming profiles can be attained by selectively activating and deactivating specific capillary devices. This concept can be further developed into modular acousto-capillary fluidics, where multiple capillary-based fluidics can perform individually specialized sequences.

The current study demonstrates liquid pumping only in one direction. Achieving bi-directional motion, i.e., infusing and retracting liquid, could benefit various microfluidics and liquid manipulation methods. The RAEE could provide bi-directional pumping by controlling the direction of the generated vortices, which can be accomplished by tuning the exciting acoustic frequency. Alternately, manipulating the acoustofluidic device towards the bottom wall can switch the direction of pumping. Consequently, the RAEE offers an easy and versatile alternative to current liquid pumps. It also supports selective pumping among multiple open channels by simply manipulating the acoustofluidic device to a specific channel. Finally, we also demonstrated the trapping of a zebrafish embryo using the acoustofluidic device. We plan to expand on this by trapping a zebrafish embryo while swimming, and by incorporating computer vision for automated size-dependent sorting, which can be used in the classification or phenotyping of zebrafish embryos.

Finally, our simple acoustofluidic device, which costs under $20 to manufacture, can be connected to any commercially available robotic arm for automation and to introduce multifunctionality—although it merits note that the precision of manipulation depends on the robot arm. Nonetheless, with its ease of construction, the versatile RAEE could become valuable in basic or translational research that calls for repetitive sample preparation steps, particularly those involving micropipetting and typically performed by manual operators.

## Methods

### Experimental set-up

#### Dynamic acoustic device

The robot-assisted acoustofluidic manipulation system (Fig. 1a) consists of a robotic arm (Dorna robotics, USA) (code to control the Dorna Robotic arm is mentioned in Supplementary Data file), a piezoelectric transducer, and a glass capillary. The piezoelectric transducer (Steminc, USA) had dimensions of 7.0 × 8.0 × 0.2 mm and a resonance frequency of 240 kHz (see also Supplementary Fig. 3). The glass capillary had a 1.5 mm outer diameter at its base and was pre-pulled, creating a narrow tip of only a few micrometres in diameter at its other end. Most of the results shown in this work are obtained using a closed capillary with no liquid inside the capillary; see Supplementary Note 4 for preliminary results for a case with liquid inside capillary. The piezoelectric transducer was bonded using a two-component epoxy (UHU Plus Sofortfest) at ~2 cm above the tip of the capillary, and was connected to a function generator (Tektronix AFG 3011 C) and activated with square waves having amplitudes in the range of 0–20 volt peak to peak (*V*_PP_) and frequencies in the range of 5–300 kHz. The capillary-based acoustofluidic device was connected to the robotic arm via a custom-built 3D printed gripper and is illustrated in Fig. 1a. The robotic arm was installed adjacent to an inverted microscope on an air-dampened table. The capillary tip was immersed in a cylindrical liquid chamber containing Dragon Green fluorescent tracer particles (Bangs Laboratories, Inc.) with a mean diameter of either 2.0 or 5.0 μm, mixed with de-ionized water in a volume ratio of 1:10 (Fig. 1a). The tip was positioned at the chamber’s centre to ensure minimum boundary effects during its characterization with the program PIVlab.

#### Static acoustic device

The acoustic device was set up as described above. A static gripper replaced the robotic arm; it was custom-made and 3D printed (Form3, FormLabs) using clear resin (see also Supplementary Fig. 5).

#### Imaging

The acoustofluidic device was placed on the sample stage of an inverted microscope (Axiovert 200M, Zeiss, Leica), and images and videos were recorded using a high-sensitivity camera (CoolSNAP EZ Monochrome, Photometrics by Hamamatsu) and a high-speed camera (Chronos 1.4).

### Preparation of polydimethylsiloxane (PDMS) channel

The PDMS spiral channel used for acoustic pumping in Fig. 4d was fabricated from a 3D-printed mould. The base (Sylgard 184 Silicone Elastomer Base, Dow Europe) and the curing agent (Sylgard 184 Silicone Elastomer Curing Agent, Dow Europe) were mixed at a 10:1 volume ratio and put under vacuum for degassing. Afterwards, PDMS was poured over the mould, then put back under vacuum. Finally, the channel was heated in an oven for 2 h at 85 °C to get the final PDMS-based channel.

### Preparation of the zebrafish embryo

Zebrafish (*Danio rerio*) were maintained under a 14:10 h light/dark cycle at 28 °C^1^. Embryos from pairwise crosses of WIK wild-type fish were raised in E3 medium (5 mM NaCl, 0.17 mM KCl, 0.33 mM CaCl_2_, 0.33 mM MgSO_4_) at 28 °C. Larvae were used at 5 days post fertilization (dpf) in E3 medium. Experiments on larvae until 5 dpf do not fall under animal welfare regulations. Husbandry and housing was approved by local authorities (Kantonales Veterinäramt TV4206).

### Reporting summary

Further information on research design is available in the Nature Research Reporting Summary linked to this article.

## Supplementary information

Supplementary Information

Description of Additional Supplementary Files

Supplementary Movie 1

Supplementary Movie 2

Supplementary Movie 3

Supplementary Movie 4

Supplementary Movie 5

Supplementary Movie 6

Supplementary Movie 7

Supplementary Movie 8

Supplementary Movie 9

Supplementary Movie 10

Supplementary Movie 11

Supplementary Movie 12

Supplementary Movie 13

Supplementary Data 1

Reporting Summary

## Data Availability

Source data are available for Figs. 1–7 and Supplementary Figs. Notes and Movies in the associated Source data file. Data that support the findings of this study are available within the paper, Supplementary Information and Supplementary data files. Source data are provided with this paper.

## Source data

Source Data

## Code availability

The MATLAB code to control the DORNA robotic arm is added in Supplementary Data file 1.

## References

[1] Ober TJ, Foresti D, Lewis JA (2015). Active mixing of complex fluids at the microscale. Proc. Natl Acad. Sci. USA.

[2] Jooss VM, Bolten JS, Huwyler J, Ahmed D (2022). In vivo acoustic manipulation of microparticles in zebrafish embryos. Sci. Adv..

[3] Bachman H (2019). Open source acoustofluidics. Lab Chip.

[4] Yang S (2022). Harmonic acoustics for dynamic and selective particle manipulation. Nat. Mater..

[5] Wu Z (2019). A digital acoustofluidic pump powered by localized fluid-substrate interactions. Anal. Chem..

[6] Zhong J (2020). When robotics met fluidics. Lab Chip.

[7] Collins DJ (2015). Two-dimensional single-cell patterning with one cell per well driven by surface acoustic waves. Nat. Commun..

[8] Chen C (2021). Acoustofluidic rotational tweezing enables high-speed contactless morphological phenotyping of zebrafish larvae. Nat. Commun..

[9] Flowbot ONE. Easy automation of your liquid handling. Flow robotics. https://flow-robotics.com/products/flowbot-one/ (2022).

[10] Tecan. Freedom EVO platform. https://lifesciences.tecan.com/freedom-evo-platform (2022).

[11] Opentrons. OT-2 Liquid Handler. Opentrons Lab Automation from $5,000. https://opentrons.com/ot-2/ (2022).

[12] Opentrons. Open-source Lab Automation, under $10,000. https://opentrons.com/ (2022).

[13] ThermoFisher SCIENTIFIC. Lab robotics. https://www.thermofisher.com/in/en/home/life-science/lab-equipment/lab-automation/lab-robotics.html (2022).

[14] Burger B (2020). A mobile robotic chemist. Nature.

[15] Ozcelik A (2018). Acoustic tweezers for the life sciences. Nat. Methods.

[16] Ahmed D, Dillinger C, Hong A, Nelson BJ (2017). Artificial acousto-magnetic soft microswimmers. Adv. Mater. Technol..

[17] Dillinger C, Nama N, Ahmed D (2021). Ultrasound-activated ciliary bands for microrobotic systems inspired by starfish. Nat. Commun..

[18] Guo, X. et al. Acoustofluidic tweezers for the 3D manipulation of microparticles. in *2020 IEEE International Conference on Robotics and Automation (ICRA)* 11392–11397 (IEEE, 2020).

[19] Schrage, M., Medany, M. & Ahmed, D. Programmable control of ultrasound swarmbots through reinforcement learning. Preprint at *arXiv* https://arxiv.org/abs/2209.15393 (2022).

[20] Schaler, E. W., Ruffatto, D., Glick, P., White, V. & Parness, A. An electrostatic gripper for flexible objects. in *2017 IEEE/RSJ International Conference on Intelligent Robots and Systems (IROS)* 1172–1179 (IEEE, 2017).

[21] Shintake J, Rosset S, Schubert B, Floreano D, Shea H (2016). Versatile soft grippers with intrinsic electroadhesion based on multifunctional polymer actuators. Adv. Mater..

[22] Probst R, Shapiro B (2011). Three-dimensional electrokinetic tweezing: device design, modeling, and control algorithms. J. Micromech. Microeng..

[23] Cohen AE, Moerner WE (2005). Method for trapping and manipulating nanoscale objects in solution. Appl. Phys. Lett..

[24] Wright SE, Mahoney AW, Popek KM, Abbott JJ (2017). The spherical-actuator-magnet manipulator: a permanent-magnet robotic end-effector. IEEE Trans. Robot..

[25] Bausch AR, Möller W, Sackmann E (1999). Measurement of local viscoelasticity and forces in living cells by magnetic tweezers. Biophys. J..

[26] Zhang P (2020). Acoustic streaming vortices enable contactless, digital control of droplets. Sci. Adv..

[27] Yang K, Peretz-Soroka H, Liu Y, Lin F (2016). Novel developments in mobile sensing based on the integration of microfluidic devices and smartphones. Lab Chip.

[28] Erickson D (2014). Smartphone technology can be transformative to the deployment of lab-on-chip diagnostics. Lab Chip.

[29] Liu X (2019). Multifunctional noncontact micromanipulation using whirling flow generated by vibrating a single piezo actuator. Small.

[30] Nilsson A, Petersson F, Jönsson H, Laurell T (2004). Acoustic control of suspended particles in micro fluidic chips. Lab Chip.

[31] Ding X (2012). On-chip manipulation of single microparticles, cells, and organisms using surface acoustic waves. Proc. Natl Acad. Sci. USA.

[32] Zhou Q, Sariola V, Latifi K, Liimatainen V (2016). Controlling the motion of multiple objects on a Chladni plate. Nat. Commun..

[33] Doinikov AA, Gerlt MS, Dual J (2020). Acoustic radiation forces produced by sharp-edge structures in microfluidic systems. Phys. Rev. Lett..

[34] Janiak, J., Doinikov, A. & Ahmed, D. Microbubble propulsion, train-like assembly and cargo transport in an acoustic field. *Lab Chip*10.1039/C4LC01266F(2022). 10.1038/s41467-023-40387-7 PMC10404234 37543657

[35] Ahmed D (2016). Rotational manipulation of single cells and organisms using acoustic waves. Nat. Commun..

[36] Catarino SO (2014). Piezoelectric actuators for acoustic mixing in microfluidic devices—numerical prediction and experimental validation of heat and mass transport. Sens. Actuators B Chem..

[37] Läubli NF (2019). 3D manipulation and imaging of plant cells using acoustically activated microbubbles. Small Methods.

[38] Baudoin M (2020). Spatially selective manipulation of cells with single-beam acoustical tweezers. Nat. Commun..

[39] Laurell, T. & Lenshof, A. *Microscale Acoustofluidics* (Royal Society of Chemistry, 2014).

[40] Yang Y (2022). Self-adaptive virtual microchannel for continuous enrichment and separation of nanoparticles. Sci. Adv..

[41] Fonseca ADC, Kohler T, Ahmed D (2022). Ultrasound-controlled swarmbots under physiological flow conditions. Adv. Mater. Interf..

[42] Tian Z (2019). Wave number–spiral acoustic tweezers for dynamic and reconfigurable manipulation of particles and cells. Sci. Adv..

[43] Garciamendez-Mijares CE, Agrawal P, Martinez GG, Juarez EC, Zhang YS (2021). State-of-art affordable bioprinters: a guide for the DiY community. Appl. Phys. Rev..

[44] Wang J (2019). Microfluidic cap-to-dispense (μCD): a universal microfluidic-robotic interface for automated pipette-free high-precision liquid handling. Lab Chip.

[45] Li J, Ha NS, Liu T, Leo, van Dam RM, CJ Kim CJ (2019). Ionic-surfactant-mediated electro-dewetting for digital microfluidics. Nature.

[46] Zhang SP (2018). Digital acoustofluidics enables contactless and programmable liquid handling. Nat. Commun..

[47] Zhu H (2021). Acoustohydrodynamic tweezers via spatial arrangement of streaming vortices. Sci. Adv..

[48] Bian S (2017). High-throughput in situ cell electroporation microsystem for parallel delivery of single guide RNAs into mammalian cells. Sci. Rep..

[49] Nama N, Huang PH, Huang TJ, Costanzo F (2014). Investigation of acoustic streaming patterns around oscillating sharp edges. Lab Chip.

[50] Sadhal SS (2012). Acoustofluidics 16: acoustics streaming near liquid–gas interfaces: drops and bubbles. Lab Chip.

[51] Läubli NF (2021). 3D mechanical characterization of single cells and small organisms using acoustic manipulation and force microscopy. Nat. Commun..

[52] Läubli NF (2021). Embedded microbubbles for acoustic manipulation of single cells and microfluidic applications. Anal. Chem..

[53] Berthier E, Beebe DJ (2007). Flow rate analysis of a surface tension driven passive micropump. Lab Chip.

[54] Hossan M, Dutta D, Islam N, Dutta P (2018). Electric field driven pumping in microfluidic device. Electrophoresis.

[55] Zhang S, Wang Y, Lavrijsen R, Onck PR, den Toonder JMJ (2018). Versatile microfluidic flow generated by moulded magnetic artificial cilia. Sens. Actuators B Chem..

[56] Maruo S, Inoue H (2007). Optically driven viscous micropump using a rotating microdisk. Appl. Phys. Lett..

[57] Tan Z, Yang M, Ripoll M (2019). Microfluidic pump driven by anisotropic phoresis. Phys. Rev. Appl..

[58] Liu F, Kc P, Zhang G, Zhe J (2017). In situ single cell detection via microfluidic magnetic bead assay. PLoS ONE.

[59] Ozcelik A, Aslan Z (2021). A practical microfluidic pump enabled by acoustofluidics and 3D printing. Microfluid Nanofluid.

[60] Perry SL, Higdon JJL, Kenis PJA (2010). Design rules for pumping and metering of highly viscous fluids in microfluidics. Lab Chip.

[61] Hanasoge S, Hesketh PJ, Alexeev A (2018). Microfluidic pumping using artificial magnetic cilia. Microsyst. Nanoeng..

[62] Lee Y-S, Bhattacharjee N, Folch A (2018). 3D-printed Quake-style microvalves and micropumps. Lab Chip.

[63] Jeong GS (2014). Siphon-driven microfluidic passive pump with a yarn flow resistance controller. Lab Chip.

[64] Chen Z, Noh S, Prisby RD, Lee J-B (2020). An implanted magnetic microfluidic pump for in vivo bone remodeling applications. Micromachines.

[65] McKnight TE, Culbertson CT, Jacobson SC, Ramsey JM (2001). Electroosmotically induced hydraulic pumping with integrated electrodes on microfluidic devices. Anal. Chem..

[66] Leach J, Mushfique H, Di Leonardo R, Padgett M, Cooper J (2006). An optically driven pump for microfluidics. Lab Chip.

[67] Shi J (2009). Acoustic tweezers: patterning cells and microparticles using standing surface acoustic waves (SSAW). Lab Chip.

[68] Tian Z (2020). Generating multifunctional acoustic tweezers in Petri dishes for contactless, precise manipulation of bioparticles. Sci. Adv..

[69] Chung K (2011). A microfluidic array for large-scale ordering and orientation of embryos. Nat. Methods.

[70] Levario TJ, Zhan M, Lim B, Shvartsman SY, Lu H (2013). Microfluidic trap array for massively parallel imaging of Drosophila embryos. Nat. Protoc..

[71] Yang Z (2019). Light sheet microscopy with acoustic sample confinement. Nat. Commun..

[72] Favre-Bulle IA (2020). Sound generation in zebrafish with bio-opto-acoustics. Nat. Commun..

[73] You JB (2019). Live sperm trap microarray for high throughput imaging and analysis. Lab Chip.

[74] Chung K, Crane MM, Lu H (2008). Automated on-chip rapid microscopy, phenotyping and sorting of *C. elegans*. Nat. Methods.

[75] Chakraborty, C., Hsu, C. H., Wen, Z. H., Lin, C. S. & Agoramoorthy, G. Zebrafish: a complete animal model for in vivo drug discovery and development. *Curr. Drug Metab.* **10**, 116–124 (2009). 10.2174/138920009787522197 19275547

[76] Miller DL (1988). Particle gathering and microstreaming near ultrasonically activated gas‐filled micropores. J. Acoust. Soc. Am..

[77] Wang S, Huang X, Yang C (2011). Mixing enhancement for high viscous fluids in a microfluidic chamber. Lab Chip.

[78] Orbay S (2016). Mixing high-viscosity fluids via acoustically driven bubbles. J. Micromech. Microeng..

[79] Huang P-H (2015). An acoustofluidic sputum liquefier. Lab Chip.

[80] du Chatinier DN, Figler KP, Agrawal P, Liu W, Zhang YS (2021). The potential of microfluidics-enhanced extrusion bioprinting. Biomicrofluidics.

[81] Guo F (2015). Controlling cell–cell interactions using surface acoustic waves. Proc. Natl Acad. Sci. USA.

[82] Li S (2014). Standing surface acoustic wave based cell coculture. Anal. Chem..

