# Supplementary material for: A robot-assisted acoustofluidic end effector
Source: Nat Commun. 2022 Oct 26;13:6370. doi: 10.1038/s41467-022-34167-y (PMC9605990; doi:10.1038/s41467-022-34167-y)
Supplement: Supplementary file 1 — Supplementary Information [file 41467_2022_34167_MOESM1_ESM.pdf]

Supplementary Information

A Robot-assisted Acoustofluidic End Effector

Jan Durrer[1], Prajwal Agrawal[1], Ali Ozgul[1], Stephan C. F. Neuhauss[2], Nitesh Nama[3] and Daniel Ahmed[1*]

[1]Acoustic Robotics Systems Lab, Institute or Robotics and Intelligent Systems, Department of Mechanical and Process Engineering, ETH Zurich, Switzerland

[2]Department of Molecular Life Sciences, University of Zurich, Switzerland

[3]Department of Mechanical & Materials Engineering, University of Nebraska-Lincoln, USA

*Correspondence and requests for materials should be addressed to D.A. (e-mail: dahmed@ethz.ch)

This PDF file includes:

Supplementary Notes
Supplementary Figures
Supplementary References

Supplementary Notes

Supplementary Note 1: Theory

In this section, we present the theoretical formulation employed in the current work to investigate the flow patterns around the oscillating tip in our system. This formulation employs the standard perturbation approach that has been employed to study acoustic streaming patterns around oscillating solid boundaries in numerous prior reports [1-7]. For the sake of completeness, we reproduce the governing equations below.

The fluid response is governed by the standard Navier-Stokes equations for a linear, viscous compressible fluid:

$$\frac{\partial \rho}{\partial t} + \nabla \cdot (\rho \mathbf{v}) = 0 , \quad (1)$$

$$\rho \frac{\partial \mathbf{v}}{\partial t} + \rho(\mathbf{v} \cdot \nabla)\mathbf{v} = -\nabla p + \mu \nabla^2 \mathbf{v} + (\mu_b + \frac{1}{3}\mu)\nabla(\nabla \cdot \mathbf{v}), \quad (2)$$

where ρ is the mass density of the fluid, p is the fluid pressure, and μ and μ_b are the shear and the bulk dynamic viscosities, respectively. These governing equations are further supplemented with a constitutive relation linking the pressure to the fluid density. Following our prior work [1,2], we assume a linear relation between pressure and density as:

$$p = c_0^2 \rho,$$

where c_0 is the speed of sound in the fluid under quiescent conditions (i.e., in absence of acoustic actuation). For high-frequency acoustic systems, the time-scale separation between the acoustic actuation and the ensuing acoustic streaming renders a direct numerical solution of these equations impractical. Therefore, we follow a perturbation approach to split the flow variables into their first- and second-order components as:

$$\mathbf{v} = \mathbf{v_0} + \varepsilon \mathbf{v_1} + \varepsilon^2 \mathbf{v_2} + O(\varepsilon^3) + \cdots ,$$

$$p = p_0 + \varepsilon p_1 + \varepsilon^2 p_2 + O(\varepsilon^3) + \cdots ,$$

$$\rho = \rho_0 + \varepsilon \rho_1 + \varepsilon^2 \rho_2 + O(\varepsilon^3) + \cdots , \quad (3)$$

where ε is a non-dimensional smallness parameter. Substitution of equation (3) in equations (1) and (2), and subsequent segregation of first-order terms yields a first-order system of equations:

$$\frac{\partial \rho_1}{\partial t} + \rho_0(\nabla \cdot \mathbf{v_1}) = 0, \tag{4}$$

$$\rho_0 \frac{\partial \mathbf{v_1}}{\partial t} = -\nabla p_1 + \mu \nabla^2 \mathbf{v_1} + (\mu_b + \frac{1}{3}\mu)\nabla(\nabla \cdot \mathbf{v_1}). \tag{5}$$

Following the same procedure for the second-order terms, combined with a time-averaging over a period of oscillation, yields the second-order system of equations:

$$\langle\frac{\partial \rho_2}{\partial t}\rangle + \rho_0 \nabla \cdot \langle \mathbf{v_2} \rangle = -\nabla \cdot \langle \rho_1 \mathbf{v_1} \rangle, \tag{6}$$

$$\rho_0 \langle\frac{\partial \mathbf{v_2}}{\partial t}\rangle + \langle \rho_1 \frac{\partial \mathbf{v_1}}{\partial t}\rangle + \rho_0 \langle(\mathbf{v_1} \cdot \nabla)\mathbf{v_1}\rangle = -\nabla p_2 + \mu \nabla^2 \mathbf{v_2} + (\mu_b + \frac{1}{3}\mu)\nabla(\nabla \cdot \mathbf{v_2}). \tag{7}$$

The above system of equations, with appropriate boundary conditions, yields the Eulerian descriptions of fluid velocity and pressure at the first- and second-order.

Supplementary Note 2: Tip streaming simulations for different oscillations

To investigate the acoustic streaming patterns caused by different oscillation profiles of the capillary tip, we performed numerical simulations using the theoretical framework described above. To this end, we considered the capillary tip to be a rigid oscillating cylinder with radius $a = 10\ \mu\mathrm{m}$. The surrounding fluid domain is taken to be large enough (radius = 100a) to avoid the effect of any boundaries on the oscillation profile near the tip. Additionally, the fluid domain is surrounded by the perfectly matching layer (PML) boundary that minimizes the acoustic reflections from the boundary.

The first-order acoustic fields are assumed to be time-harmonic, and therefore a harmonic solution is sought for equations (4) and (5). Here, the tip boundary is prescribed a Dirichlet boundary condition with a known displacement (or velocity) profile. Once the first-order flow fields are solved for, the source terms in second-order equations (6) and (7) are identified and a steady solution is sought for the second-order fields. For the second-order problem, a Dirichlet boundary condition is prescribed for the Lagrangian velocity at the tip boundary. We refer the reader to prior reports [1,2,6,7] for further discussion of the governing equations and numerical solution strategies.

Supplementary Fig. 1 presents the numerically obtained acoustic streaming profiles around the oscillating tip for various oscillation profiles. Specifically, we prescribed three different oscillation profiles of the capillary tip: (a) rectilinear oscillations purely along Y direction, (b) oscillations along both X and Y direction with a phase difference of $\pi/4$, and (c) oscillations along both X and Y direction with a phase difference of $\pi/2$. As expected, the oscillation profile can be seen to have a significant effect on the acoustic streaming patterns. Since the capillary tip oscillation profile varies with the actuation frequency (see Fig. 2(g) and (h) in the main text), these numerical results indicate that different oscillation patterns observed in Fig. 2(a-f) are a consequence of the frequency-dependent tip oscillation profiles.

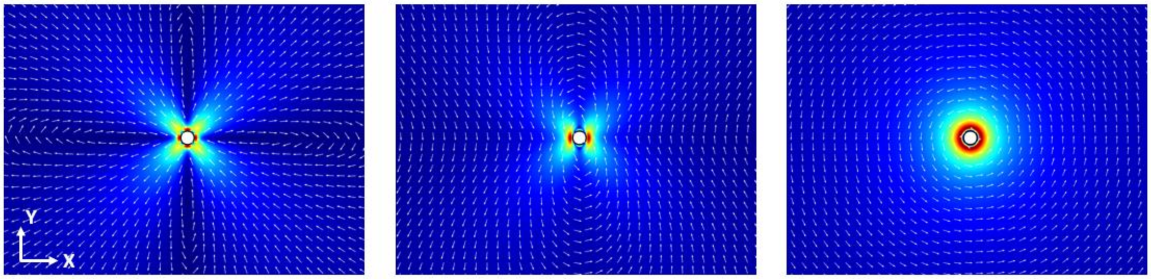

Supplementary Fig. 1| Acoustic streaming patterns around the oscillating capillary tip for different oscillation profiles: (a) rectilinear oscillations purely along Y direction, (b) oscillations along both X and Y direction with a phase difference of $\pi/4$, and (c) oscillations along both X and Y direction with a phase difference of $\pi/2$. Colors indicate the magnitude of streaming velocity ranging from blue (minimum) to red (maximum), while the arrows indicate the direction of the (normalized) streaming velocity. The numerical values of the streaming velocity depend on the displacement amplitude of the tip.

Supplementary Note 3: Governing equations for trapping potential of microparticles

The total acoustic force on a spherical particle in our system can be described as [8,9]

$$\mathbf{F}_{\mathrm{rad}} = \int_{S_0} [< \boldsymbol{\sigma}_2 > -\rho_0 < \mathbf{v}_1\mathbf{v}_1 >] \cdot \mathbf{n}_0 \, dS \qquad (8)$$

where $<\cdot>$ denotes the time average, $\boldsymbol{\sigma}_2$ represents the second-order stress tensor, ρ_0 represents the fluid density in unperturbed state, $\mathbf{v}_1$ represents the first-order acoustic velocity, S_0 represents an arbitrary static surface enclosing the particle, and $\mathbf{n}_0$ represents the corresponding outward pointing unit normal. Therefore, the term inside the brackets represents the difference between the mean second-order stress tensor and Reynold stress.

This expression can be viewed as a combination of the forces arising from three contributions: (1) hydrodynamic forces arising from the bulk acoustic streaming (that is generated irrespective of the presence of particle), (2) hydrodynamic forces arising from the acoustic microstreaming (i.e., the acoustic streaming around a solid surface, generated solely due to the presence of solid surface in a viscous fluid), and (3) the Reynolds stress tensor arising from the nonlinear interactions of first order fields. We remark that the above expression includes the viscous effects at second-order, that are contained with the stress tensor σ_2.

Further, we note that depending on the particle size in relation to the viscous boundary layer thickness, the contribution of microstreaming to the total acoustic radiation force may or may not be significant. Specifically, in the regime considered in the current article (frequencies ~100 kHz), the boundary layer thickness (δ) is of the order of 1 µm. If a particle size larger than boundary layer thickness is considered, (i.e., $\frac{\delta}{a} < 1$)., the viscous effects at the second-order can be neglected and the acoustic radiation force can be expressed as [8]

$$\mathbf{F}_{\mathrm{rad}} = -\pi a^3 \left[\frac{2\kappa_0}{3} \mathrm{Re}[f_1^* p_1^* \nabla p_1] - \rho_0 \mathrm{Re}[f_2^* \mathbf{v}_1^* \cdot \nabla \mathbf{v}_1]\right] \qquad (9)$$

where p_1 and $\mathbf{v}_1$ are the first-order pressure and velocity fields, κ_0 is the compressibility of the liquid, $\mathrm{Re}[\cdot]$ denotes the real part of a quantity, the asterix denotes complex conjugates, and f_1 and f_2 are coefficients given as

$$f_1 = 1 - \frac{\kappa_p}{\kappa_0} \qquad (10)$$

$$f_2 = \frac{2(\rho_p - \rho_0)}{2\rho_p + \rho_0} \qquad (11)$$

Supplementary Note 4: Acoustic effects inside the glass capillary

The capillary used in the main text is a closed capillary, meaning that no fluid is present within the capillary during the experiment. Furthermore, both closed and open capillaries produced the same results in the experiments. Nevertheless, the use of an open capillary allows the pumping of fluid for various purposes, such as microinjection, liquid or particle transport, etc. The results of the effect of fluid inside the capillary are illustrated in **Supplementary Fig. 2.** As a result of oscillations at the capillary tip, this induced streaming is observed in the fluid (outside) surrounding the capillary. Further analysis was performed on the fluid inside the capillary that resided in a wider part of the capillary and was not pulled to a narrow and sharp tip. Due to the lack of fluid circulation within the capillary, it is assumed that the oscillating tip of the capillary only affected fluid located outside the capillary. Upon analyzing the results of the experiments, it was concluded that the acoustic effects can also be felt inside the capillaries. During the experiment, particles (beads) of various sizes were used inside the capillary. Upon activation of the transducer, particles separated according to their size. In addition, we were able to create multiple nodes in which particles were trapped. In order to move the particles reliably, the acoustic frequency was changed to reversibly move the nodes along the longitudinal axis of the capillaries after the particles had been trapped.

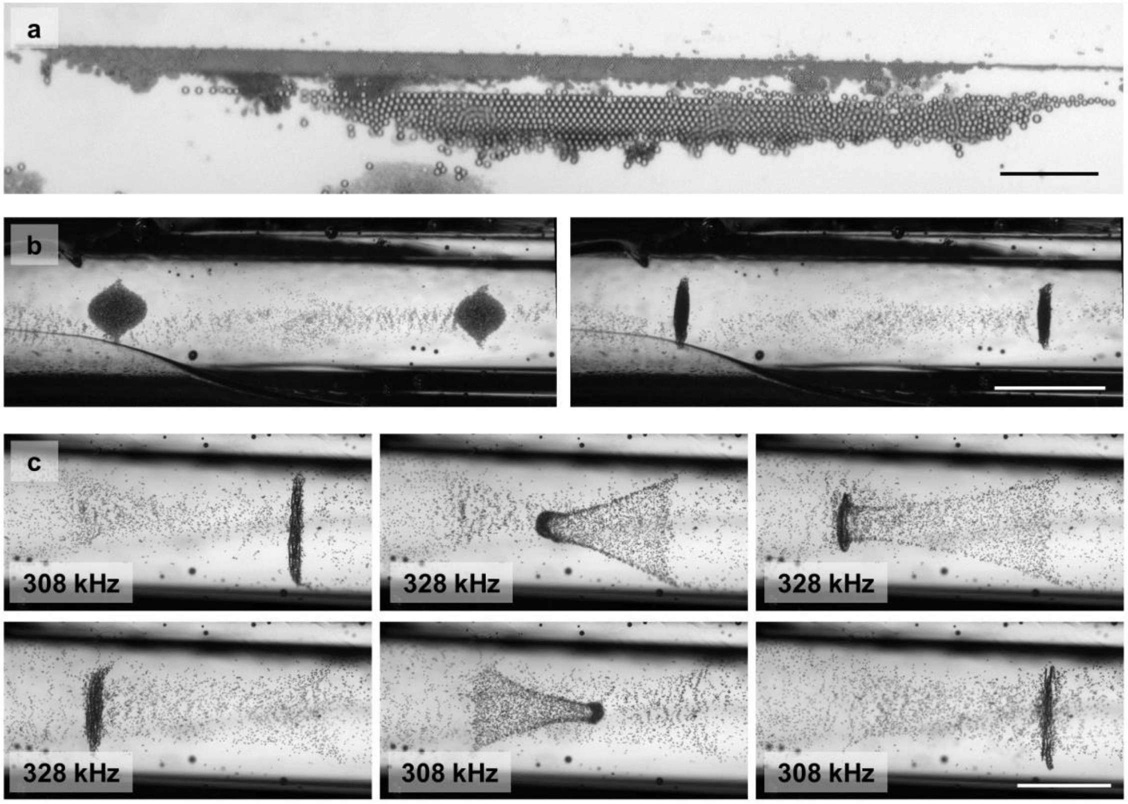

Supplementary Fig. 2| Acoustic phenomenon inside the glass capillary. **a.** Tracer particles of the size 2 and 6 µm were separated depending on their size inside the glass capillary using acoustic waves at excitation frequency and amplitude of $\mathbf{308\ kHz}$ and $\mathbf{5\ V_{PP}}$, respectively. Scale bar: 100 µm. **b.** Tracer particles were trapped at the nodes of the acoustic waves at excitation frequency $\mathbf{308\ kHz}$ and $\mathbf{5\ V_{PP}}$, respectively. Left: Acoustics off. Right: Acoustics on. Scale bar: 1mm. **c.** The position of the acoustic nodes could be shifted when the frequency of the wave was changed. The

node at $\mathbf{308\ kHz}$ could be shifted to the left at $\mathbf{328\ kHz}$ and shifted back at $\mathbf{308\ kHz}$. The applied amplitude was kept constant at $\mathbf{5\ V_{PP}}$. Scale bar: 1mm

Supplementary Figures:

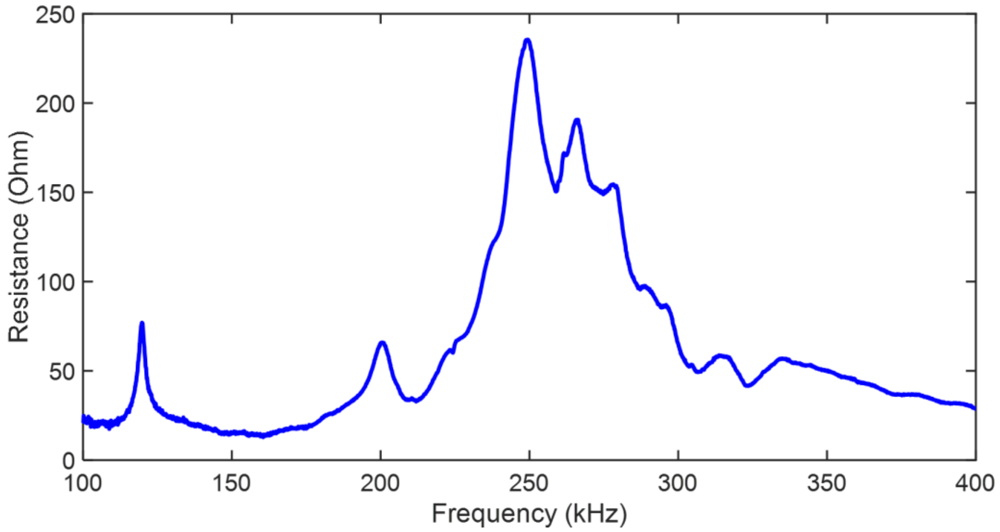

Supplementary Fig. 3| Impedance measurement (Real part of impedance = resistance) of the piezo transducer coupled with a glass capillary using an impedance analyzer (SinePhase, Impedance Analyzer 16777k). The experimental setup showed resonances at $\sim 249.4\ \mathrm{kHz}$. See Supplementary data file 5 for the source data for the graph.

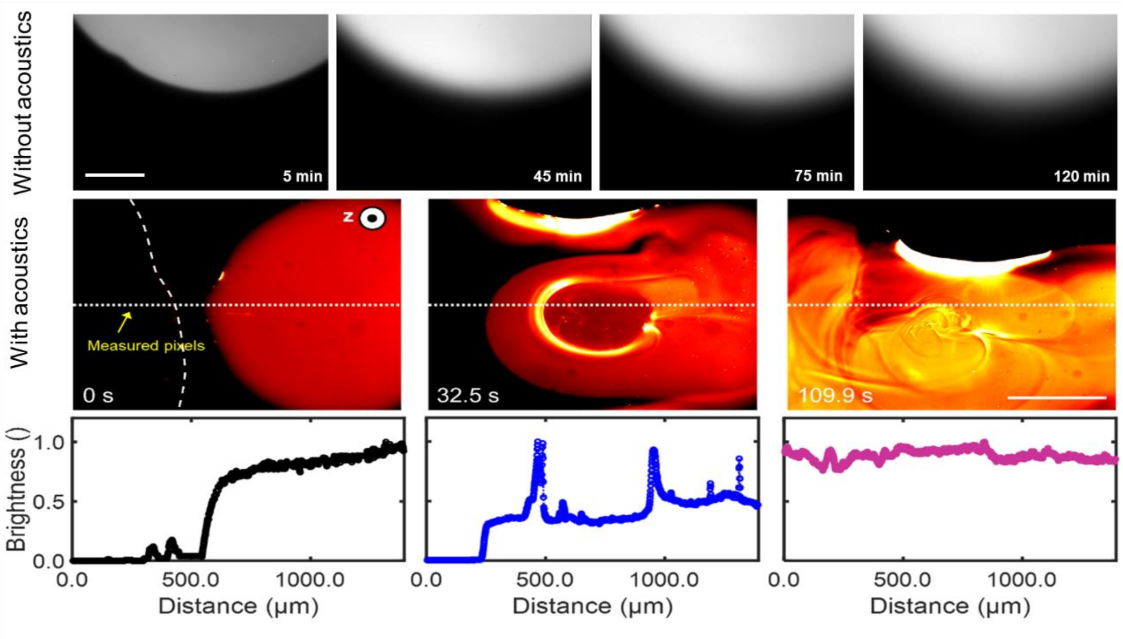

Supplementary Fig. 4| (Top panel) Image sequences demonstrate negligible diffusion-based mixing between glycerol and rhodamine droplets. (Middle panel) In the left image, where the initial position $(0s)$ is depicted, the two separated droplets were not yet merged. The right, red droplet (rhodamine mixed in water) is where the acoustofluidic device connected to the robotic arm with a precision of 0.02 mm repeatability is initially located. The boundary of the left droplet (glycerol) was indicated by a white dashed line. In the intensity plot, the unmerged state is recognizable at the flat line close to the zero axis in the left of the intensity plot. Once the red droplet appears in the image, the intensity line resembles a step function, indicating a sharp edge between two unmixed regions. In the middle image $(32.5s)$ The previously separated droplets were merged using the acoustofluidic device in combination with the robotic arm. The intensity plot depicts a step function corresponding to the location where we recognize the border between the red and black areas along the dotted line in the microscope image, where the mixing of the two droplets is currently occurring. In the rightmost image $(109.9s)$ all pixel intensities are close to the highest measured value and no step function can be seen, which we can validate by comparing the intensity plot to the microscope image. The two droplets were well mixed. (Bottom panel) Viscous mixing efficiency analysis. The intensity plot along the indicated line in the recorded footage was analyzed. The intensity of each image was normalized by the highest occurring intensity of the respective frame. See Supplementary data file 6 for the source data for the graphs.

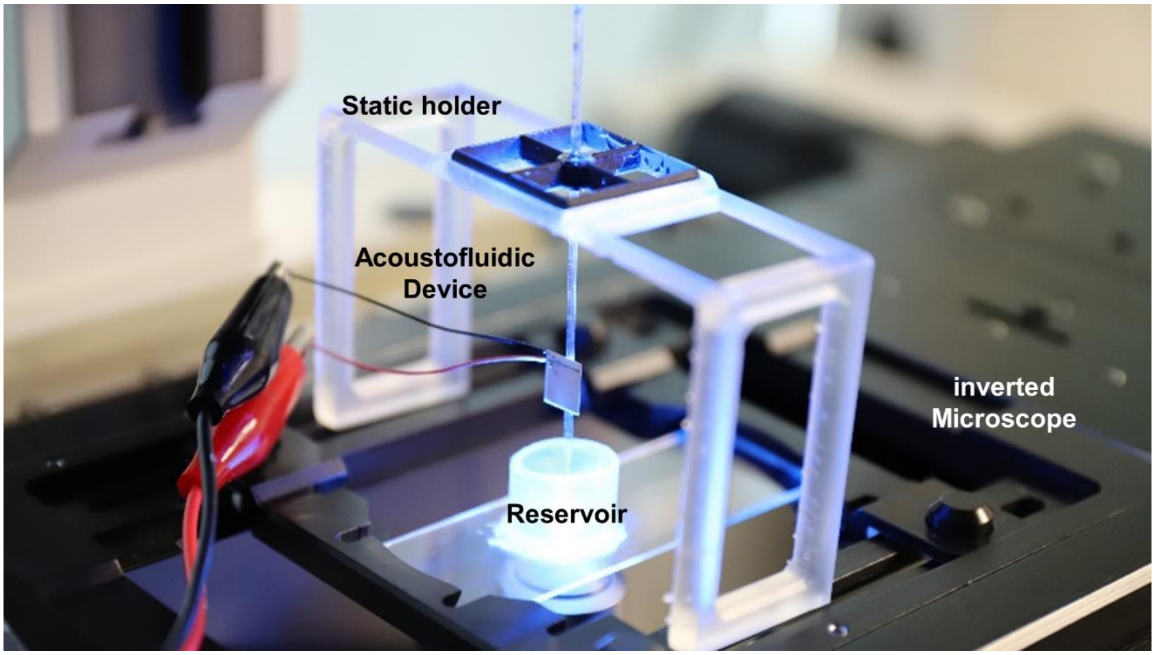

Supplementary Fig. 5| Acousticfluidic setup. The acoustofluidic system was built around a $25 \text{ mm x } 75 \text{ mm x } 1 \text{ mm}$ glass slide (Menzel) whereon a 3D printed liquid reservoir was attached with epoxy resin (2-K-Epoxidkleber, UHU Schnellfest). Once the acoustofluidic device and the static holder were fabricated and cleaned (IPA, Sigma-Aldrich), they were transferred onto the microscope stage. The acoustofluidic device consisted of a piezo transducer (Steminc, USA) connected to a pre-pulled glass capillary using epoxy resin (2-K-Epoxidkleber, UHU Schnellfest). The piezo with a size of $7 \text{ x } 8 \text{ x } 0.2 \text{ mm}$ had a corresponding resonance of 240 kHz. The glass capillary with aouter diameter of 1.5 mm was pulled by clamped at both ends, heated up in the middle and pulled outwards at the clamps, resulting in a long, tailored, narrow tip of less than 10 μm diameter. A solution consisting of $10{:}\,1$ by volume DI water and $2.07 \text{ } \mu\text{m}$ fluorescent tracer particles (FSDG005, Bangs Laboratories, Inc.) was placed into the reservoir $(\sim 200 \text{ } \mu\text{l})$. The piezo transducer was then connected to the function generator (AFG 3011C, Tektronix) to generate acoustic fields with adjustable frequencies and voltages from 0-to-20-volt peak to peak (VPP) within the liquid. The whole setup was mounted on an inverted microscope.

Supplementary References

1. Nama, Nitesh, et al. “Investigation of acoustic streaming patterns around oscillating sharp edges.” *Lab on a Chip*14.15 (2014): 2824-2836.
2. Nama, Nitesh, et al. “Investigation of micromixing by acoustically oscillated sharp-edges.” *Biomicrofluidics* 10.2 (2016): 024124.
3. Dillinger, Cornel, Nitesh Nama, and Daniel Ahmed. “Ultrasound-activated ciliary bands for microrobotic systems inspired by starfish.” *Nature communications* 12.1 (2021): 1-11.
4. Sadhal, S. S. “Acoustofluidics 13: Analysis of acoustic streaming by perturbation methods.” *Lab on a Chip* 12.13 (2012): 2292-2300.
5. Ding, Xiaoyun, et al. “Surface acoustic wave microfluidics.” *Lab on a Chip* 13.18 (2013): 3626-3649.
6. Muller, Peter Barkholt, et al. “A numerical study of microparticle acoustophoresis driven by acoustic radiation forces and streaming-induced drag forces.” *Lab on a Chip* 12.22 (2012): 4617-4627.
7. Nama, Nitesh, Tony Jun Huang, and Francesco Costanzo. “Acoustic streaming: an arbitrary Lagrangian–Eulerian perspective.” *Journal of fluid mechanics* 825 (2017): 600-630.
8. Karlsen, Jonas T., and Henrik Bruus. “Forces acting on a small particle in an acoustical field in a thermoviscous fluid.” *Physical Review E* 92.4 (2015): 043010.
9. Baasch, Thierry, Alen Pavlic, and Jürg Dual. “Acoustic radiation force acting on a heavy particle in a standing wave can be dominated by the acoustic microstreaming.” *Physical Review E* 100.6 (2019): 061102.
