# Supplementary material for: A robot-assisted acoustofluidic end effector
Source: Nat Commun. 2022 Oct 26;13:6370. doi: 10.1038/s41467-022-34167-y (PMC9605990; doi:10.1038/s41467-022-34167-y)
Supplement: Supplementary file 2 — Description of Additional Supplementary Files [file 41467_2022_34167_MOESM2_ESM.pdf]

Description of Additional Supplementary Files

Supplementary Movie 1. Programmed movement of the 5-axis Dorna robotic arm.

Supplementary Movie 2. Acoustic out-of-plane streaming around the pulled glass capillary at excitation frequency and amplitude of $\mathbf{207\ kHz}$ and $\mathbf{5\ V_{PP}}$, respectively.

Supplementary Movie 3. Tracer particles travelling around the tailoring acoustofluidic device while acoustics is applied at an excitation frequency and amplitude of $\mathbf{240\ kHz}$ and $\mathbf{2.5\ V_{PP}}$, respectively. The particles group up at equally spaced positions along the capillary.

Supplementary Movie 4. Acoustic sharp edge streaming at the tip of the pulled glass capillary at different excitation frequencies and amplitude of $\mathbf{1\text{–}3\ V_{PP}}$, respectively.

Supplementary Movie 5. Acoustic activated tip movement in air at excitation frequency and amplitude of $\mathbf{6.8\ kHz}$ and $\mathbf{20\ V_{PP}}$ (top), and $\mathbf{7.8\ kHz}$ and $\mathbf{20\ V_{PP}}$ (bottom), respectively.

Supplementary Movie 6. Trajectory control of the in action acoustofluidic device using the robotic arm. Acoustic circular streaming profile of the acoustofluidic device at excitation frequency and amplitude of $\mathbf{140\ kHz}$ and $\mathbf{1.3\ V_{PP}}$, respectively.

Supplementary Movie 7. Particle trapping of 2, 10, and 15 microns polystyrene particles around glass capillary.

Supplementary Movie 8. Acoustic activated capillary movement in air at excitation frequency and amplitude of $\mathbf{200\ kHz}$ and $\mathbf{20\ V_{PP}}$. The oscillation of the capillary resembles a standing wave with a wavelength of $\boldsymbol{\lambda} = \sim\mathbf{90\ \mu m.}$

Supplementary Movie 9. Attracting and grabbing of a zebrafish larva by trapping the swim bladder at the sharp tip of the glass capillary while applying acoustics. The zebrafish larva is released once the acoustics are turned off.

Supplementary Movie 10. The acoustofluidic device as a liquid micropump, creating a fluid flow in a spiral PDMS microchannel. The acoustofluidic device was activated at excitation frequency and amplitude of $\mathbf{134\ kHz}$ and $\mathbf{10\ V_{PP}}$, respectively.

Supplementary Movie 11. Droplet stretching and merging of two separated droplets using the acoustofluidic device in combination with the robotic arm at excitation frequency and amplitude of $\mathbf{44.9\ kHz}$ and $\mathbf{20\ V_{PP}}$, respectively.

Supplementary Movie 12. Droplet viscous mixing using the acoustofluidic device in combination with the robotic arm at excitation frequency and amplitude of $\mathbf{44.9\ kHz}$ and $\mathbf{20\ V_{PP}}$, respectively.

Supplementary Movie 13. High throughput viscous mixing of a 96-well plate using acoustics and a robotic arm at excitation frequency and amplitude of $\mathbf{240\ kHz}$ and $\mathbf{20\ V_{PP}}$, respectively.

Supplementary Data 1: MATLAB Code to control the Dorna Robotic Arm.
