# Supplementary material for: A robot-assisted acoustofluidic end effector
Source: Nat Commun. 2022 Oct 26;13:6370. doi: 10.1038/s41467-022-34167-y (PMC9605990; doi:10.1038/s41467-022-34167-y)
Supplement: Supplementary file 16 — Supplementary Data 1 [file 41467_2022_34167_MOESM16_ESM.pdf]

Program code

```
from dorna import Dorna
import math
import numpy as np

robot.connect(port_name = None)
print(robot.homed())

### -------------------------------------------------------------------- ###
###                    ROBOT CONTROL                                     ###
### -------------------------------------------------------------------- ###

def homing(robot):
    '''
    Initiate the Hall Sensors.
    robot [Dorna Object]: pass robot object for controls
    '''
    robot.home("j0")
    robot.home("j2")
    robot.home("j1")
    robot.home("j3")
    print(robot.homed())

def set_joint(robot):
    '''
    Initiate the Hall Sensors by manually setting the joints.
    robot [Dorna Object]: pass robot object for controls
    '''
    position = robot.position("joint")
    robot.set_joint({"j0": position[0]})
    robot.set_joint({"j1": position[1]})
    robot.set_joint({"j2": position[2]})
    robot.set_joint({"j3": position[3]})
    print(robot.homed())

def microscope(robot):
    '''
    Move the acoustic device above the microscope.
    robot [Dorna Object]: pass robot object for controls
    '''
    robot.play({"command":"move","prm":{"path":"joint","movement":0,"speed":1000,"j0":-
8.27,"j1":139.73,"j2":-87.18,"j3":0,"j4":0},"fulfill":False})

robot.play({"command":"move","prm":{"path":"joint","movement":0,"speed":1000,"j0":75,"j1":139.73,"j2":-
87.18,"j3":-30,"j4":0},"fulfill":False})
    robot.play({"command":"move","prm":{"path":"joint","movement":0,"speed":1000,"j1":70.26,"j2":-
59.65,"j3":-30,"j4":0},"fulfill":False})

robot.play({"command":"move","prm":{"path":"joint","movement":0,"speed":1000,"j0":90.22},"fulfill":False})

# Move the capillary tip in an "oo" inside the well of the wellplate
def mix(robot, speed = 30, ratiox = 1, ratioy = 1):
    '''
    "Lying 8" movement of the acoustic device inside the well to mix.
```

```
    robot [Dorna Object]: pass robot object for controls
    '''
    for _ in range(30):
        move_xyz(robot, 0.5, 0.5, 0, speed = speed, ratiox = ratiox, ratioy = ratioy)
        move_xyz(robot, 0.5, -0.5, 0, speed = speed, ratiox = ratiox, ratioy = ratioy)
        move_xyz(robot, -0.5, -0.5, 0, speed = speed, ratiox = ratiox, ratioy = ratioy)
        move_xyz(robot, -0.5, 0.5, 0, speed = speed, ratiox = ratiox, ratioy = ratioy)

        move_xyz(robot, -0.5, 0.5, 0, speed = speed, ratiox = ratiox, ratioy = ratioy)
        move_xyz(robot, -0.5, -0.5, 0, speed = speed, ratiox = ratiox, ratioy = ratioy)
        move_xyz(robot, 0.5, -0.5, 0, speed = speed, ratiox = ratiox, ratioy = ratioy)
        move_xyz(robot, 0.5, 0.5, 0, speed = speed, ratiox = ratiox, ratioy = ratioy)

def wellplate(robot, wells_x_dir = 3, wells_y_dir = 4, speed = 1000, ratiox = 1, ratioy = 0.96):
    '''
    Mixing of Chemicals in a 96-Wellplate using the acoustic device.
    robot [Dorna Object]: pass robot object for controls
    wells_x_dir [int]: amount of wells to mix in x-direction (Columns, Number 1-12)
    wells_y_dir [int]: amount of wells to mix in y-direction (Rows, Letters A-H)
    '''
    #check for entry error
    if wells_x_dir <= 0:
        print("wells_x_dir must be bigger than zero!")
        return

    if wells_y_dir <= 0:
        print("wells_y_dir must be bigger than zero!")
        return

    # distance from well center to well center in x or y direction
    dist_center = -9

    # move from well to well
    for columns in range(wells_x_dir):
        if (columns+1) % 2:
            for rows in range(wells_y_dir-1):
                move_z(robot, -10)
                mix(robot, speed = speed)
                move_z(robot, 10)
                move_y(robot, dist_center, ratioy = ratioy)
        else:
            for rows in range(wells_y_dir-1):
                move_z(robot, -10)
                mix(robot, speed = speed)
                move_z(robot, 10)
                move_y(robot, -dist_center, ratioy = ratioy)

        # mix and move to next column
        move_z(robot, -10)
        mix(robot, speed = speed)
        move_z(robot, 10)
        move_x(robot, -dist_center, ratiox = ratiox)

    # move back to start position
    if wells_x_dir % 2:
        for rows in range(wells_y_dir-1):
```

```
            move_y(robot, -dist_center, ratioy=ratioy)
    for columns in range(wells_x_dir):
        move_x(robot, dist_center, ratiox = ratiox)

def move_xyz(robot, x, y, z, speed = 1000, phi = 0, ratiox = 1, ratioy = 1): # phi = 0.0365
    '''
    robot [Dorna Object]: pass robot object for controls
    x [float]: distance to move in x-direction (mm)
    y [float]: distance to move in y-direction (mm)
    z [float]: distance to move in z-direction (mm)
    phi [float]: angle of rotation around the z-axis btw. the coord. sys. of the robot and the microscope (rad)
    ratiox [float]: scale distance in x-direction to adjust for errors.
    ratioy [float]: scale distance in y-direction to adjust for errors.
    '''
    x, y, z = transform(x, y, z, phi)
    x = x*ratiox
    y = y*ratioy
    robot.play({"command":"move","prm":{"path":"line","movement":1,"speed":speed,"xyz":[x,y,z]}})

def move_x(robot, distance, speed = 1000, phi = 0, ratiox = 1):
    '''
    robot [Dorna Object]: pass robot object for controls
    x [float]: distance to move in x-direction (mm)
    phi [float]: angle of rotation around the z-axis btw. the coord. sys. of the robot and the microscope (rad)
    ratiox [float]: scale distance in x-direction to adjust for errors.
    '''
    x, y, z = transform(distance, 0, 0, phi)
    x = x*ratiox

    robot.play({"command":"move","prm":{"path":"line","movement":1,"speed":speed,"xyz":[x,y,z]}})

def move_y(robot, distance, speed = 1000, phi = 0, ratioy = 1):
    '''
    robot [Dorna Object]: pass robot object for controls
    y [float]: distance to move in y-direction (mm)
    phi [float]: angle of rotation around the z-axis btw. the coord. sys. of the robot and the microscope (rad)
    ratioy [float]: scale distance in y-direction to adjust for errors.
    '''
    x, y, z = transform(0, distance, 0, phi)
    y = y*ratioy
    robot.play({"command":"move","prm":{"path":"line","movement":1,"speed":speed,"xyz":[x,y,z]}})

def move_z(robot, distance, speed = 1000):
    '''
    robot [Dorna Object]: pass robot object for controls
    z [float]: distance to move in z-direction (mm)
    '''
    robot.play({"command":"move","prm":{"path":"line","movement":1,"speed":speed,"xyz":[0,0,distance]}})

def move_j0(robot, angle):
    '''
    Rotate joint 0 relative to current configuration.
    robot [Dorna Object]: pass robot object for controls
    angle [float]: rotational angle (°)
    '''
    # angle = deg_to_rad(angle)
```

```
    robot.play({"command":"move","prm":{"path":"joint","movement":1,"speed":1000,"j0":angle}})

def move_j1(robot, angle):
    '''
    Rotate joint 1 relative to current configuration.
    robot [Dorna Object]: pass robot object for controls
    angle [float]: rotational angle (°)
    '''
    # angle = deg_to_rad(angle)
    robot.play({"command":"move","prm":{"path":"joint","movement":1,"speed":1000,"j1":angle}})

def move_j2(robot, angle):
    '''
    Rotate joint 2 relative to current configuration.
    robot [Dorna Object]: pass robot object for controls
    angle [float]: rotational angle (°)
    '''
    # angle = deg_to_rad(angle)
    robot.play({"command":"move","prm":{"path":"joint","movement":1,"speed":1000,"j2":angle}})

def move_j3(robot, angle):
    '''
    Rotate joint 3 relative to current configuration.
    robot [Dorna Object]: pass robot object for controls
    angle [float]: rotational angle (°)
    '''
    # angle = deg_to_rad(angle)
    robot.play({"command":"move","prm":{"path":"joint","movement":1,"speed":1000,"j3":angle}})

def move_j4(robot, angle):
    '''
    Rotate joint 4 relative to current configuration.
    robot [Dorna Object]: pass robot object for controls
    angle [float]: rotational angle (°)
    '''
    # angle = deg_to_rad(angle)
    robot.play({"command":"move","prm":{"path":"joint","movement":1,"speed":1000,"j4":angle}})

def drag(robot, velocity = 30):
    '''
    Drag filament from droplet.
    robot [Dorna Object]: pass robot object for controls
    '''
    robot.play({"command":"move","prm":{"path":"line","movement":1,"speed":velocity,"xyz":[0,-1.5,0]}})
    robot.play({"command":"move","prm":{"path":"line","movement":1,"speed":1000,"xyz":[0,0,0.1]}})
    robot.play({"command":"move","prm":{"path":"line","movement":1,"speed":1000,"xyz":[0,1.5,0]}})
    robot.play({"command":"move","prm":{"path":"line","movement":1,"speed":100,"xyz":[0,0,-0.1]}})

def pdms_alexia(robot):
    '''
    After homing, MOVE J3 AND J4 IN CORRECT POSITION FIRST! THEY ARE HORRIBLE TO SET
UP.
    '''
    robot.play({"command":"move","prm":{"path":"joint","movement":0,"speed":1000,"j0":86.73121}})
    robot.play({"command":"move","prm":{"path":"joint","movement":0,"speed":1000,"j2":-87.1819}})
    robot.play({"command":"move","prm":{"path":"joint","movement":0,"speed":1000,"j1":99.7334}})
```

```
    move_j3(robot, -110)

def shutdown(robot):
    '''
    Disconnect the robot and delete the Dorna Object.
    robot [Dorna Object]: pass robot object for controls
    '''
    robot.disconnect()
    robot.terminate()

### ---------------------------------------------------------------------- ###
###                    HELPER FUNCTIONS                                    ###
### ---------------------------------------------------------------------- ###


def deg_to_rad(degree):
    '''
    convert an angle in degree to radians.
    degree [float]: Angle (°)
    '''
    return degree/180*math.pi

def transform(x, y, z, phi, a=0, b=0, c=0):
    '''
    Homogeneous Transformation between the coord. sys of robot and the target.
    x, y, z [float]: distance in the robot coord. sys. (mm)
    '''
    T = np.array([[math.cos(phi), -math.sin(phi), 0, a],
                  [math.sin(phi), math.cos(phi), 0, b],
                  [0, 0, 1, c],
                  [0, 0, 0, 1]])
    B = np.array([[x],
                 [y],
                 [z],
                 [1]])
    result = np.matmul(T,B)
    x_microscope = round(float(result[0]),2)
    y_microscope = round(float(result[1]),2)
    z_microscope = round(float(result[2]),2)
    return x_microscope, y_microscope, z_microscope

def inv_transform(x, y, z, phi, a=0, b=0, c=0):
    '''
    Inversed Homogeneous Transformation between the coord. sys of robot and target.
    x, y, z[float]: distance in the target coord. sys.
    '''
    T = np.zeros((4,4))
    C = np.array([[math.cos(phi), -math.sin(phi), 0],
                  [math.sin(phi), math.cos(phi), 0],
                  [0, 0, 1]])
    A = np.array([[a],[b],[c]])
    T[0:3,0:3] = C.transpose()
    T[0:3,3:4] = -np.matmul(C.transpose(),A)
    T[3,3] = 1
    B = np.array([[x],
                 [y],
```

```
        [z],
        [1]])
result = np.matmul(T,B)
x_robot = round(float(result[0]),2)
y_robot = round(float(result[1]),2)
z_robot = round(float(result[2]),2)
return x_robot, y_robot, z_robot
```
