# Supplementary material for: A robot-assisted acoustofluidic end effector
Source: Nat Commun. 2022 Oct 26;13:6370. doi: 10.1038/s41467-022-34167-y (PMC9605990; doi:10.1038/s41467-022-34167-y)
Supplement: Supplementary file 17 — Reporting Summary [file 41467_2022_34167_MOESM17_ESM.pdf]

Corresponding author(s): Daniel Ahmed

Last updated by author(s): Sep 23, 2022

Reporting Summary

Nature Portfolio wishes to improve the reproducibility of the work that we publish. This form provides structure for consistency and transparency in reporting. For further information on Nature Portfolio policies, see our Editorial Policies and the Editorial Policy Checklist.

Statistics

For all statistical analyses, confirm that the following items are present in the figure legend, table legend, main text, or Methods section.

n/a	Confirmed	
☐	☒	The exact sample size (*n*) for each experimental group/condition, given as a discrete number and unit of measurement
☐	☒	A statement on whether measurements were taken from distinct samples or whether the same sample was measured repeatedly
☒	☐	The statistical test(s) used AND whether they are one- or two-sided<br>*Only common tests should be described solely by name; describe more complex techniques in the Methods section.*
☒	☐	A description of all covariates tested
☐	☒	A description of any assumptions or corrections, such as tests of normality and adjustment for multiple comparisons
☐	☒	A full description of the statistical parameters including central tendency (e.g. means) or other basic estimates (e.g. regression coefficient) AND variation (e.g. standard deviation) or associated estimates of uncertainty (e.g. confidence intervals)
☒	☐	For null hypothesis testing, the test statistic (e.g. *F*, *t*, *r*) with confidence intervals, effect sizes, degrees of freedom and *P* value noted<br>*Give P values as exact values whenever suitable.*
☒	☐	For Bayesian analysis, information on the choice of priors and Markov chain Monte Carlo settings
☒	☐	For hierarchical and complex designs, identification of the appropriate level for tests and full reporting of outcomes
☒	☐	Estimates of effect sizes (e.g. Cohen's *d*, Pearson's *r*), indicating how they were calculated

Our web collection on statistics for biologists contains articles on many of the points above.

Software and code

Policy information about availability of computer code

Data collection	CoolSNAP EZ Monochrome, Photometrics by Hamamatsu, and a high-speed camera (Chronos 1.4) were used to acquire images and videos from Zeiss Axiovert 200M and Leica microscopes. A cannon camera was used to image the experimental setup and other experimental data.
Data analysis	All the image processing was performed using ImageJ 1.53k (v-Java 1.8.0_172 (64-bit)).

For manuscripts utilizing custom algorithms or software that are central to the research but not yet described in published literature, software must be made available to editors and reviewers. We strongly encourage code deposition in a community repository (e.g. GitHub). See the Nature Portfolio guidelines for submitting code & software for further information.

Data

Policy information about availability of data

All manuscripts must include a data availability statement. This statement should provide the following information, where applicable:

- Accession codes, unique identifiers, or web links for publicly available datasets
- A description of any restrictions on data availability
- For clinical datasets or third party data, please ensure that the statement adheres to our policy

Source data is available for Figs. 1–7 and Supplementary Figs., Notes and Movies in the associated source data file. Data that support the findings of this study are available within the paper, Supplementary Information and Supplementary data files. The MATLAB code to control the DORNA robotic arm is added in Supplementary Data file 1.

Human research participants

Policy information about studies involving human research participants and Sex and Gender in Research.

Reporting on sex and gender	N/A
Population characteristics	N/A
Recruitment	N/A
Ethics oversight	N/A

Note that full information on the approval of the study protocol must also be provided in the manuscript.

Field-specific reporting

Please select the one below that is the best fit for your research. If you are not sure, read the appropriate sections before making your selection.

☒ Life sciences ☐ Behavioural & social sciences ☐ Ecological, evolutionary & environmental sciences

For a reference copy of the document with all sections, see nature.com/documents/nr-reporting-summary-flat.pdf

Life sciences study design

All studies must disclose on these points even when the disclosure is negative.

Sample size	The presented results are based on secondary experiments, while the first experiments were used to observe the sample variations and define sample size.
Data exclusions	No data acquired for quantitative analysis were excluded.
Replication	A minimum of three times of experiments have been conducted independently for a vast majority of conditions. The zebrafish embryo trapping experiment was reproducible.
Randomization	The imaging of all the samples was randomly allocated.
Blinding	A blind approach in this experiment was not possible since the experiments were visible. However, all the samples were collected and analyzed under the same condition.

Reporting for specific materials, systems and methods

We require information from authors about some types of materials, experimental systems and methods used in many studies. Here, indicate whether each material, system or method listed is relevant to your study. If you are not sure if a list item applies to your research, read the appropriate section before selecting a response.

Materials & experimental systems

n/a	Involved in the study	
☒	☐	Antibodies
☒	☐	Eukaryotic cell lines
☒	☐	Palaeontology and archaeology
☐	☒	Animals and other organisms
☒	☐	Clinical data
☒	☐	Dual use research of concern

Methods

n/a	Involved in the study	
☒	☐	ChIP-seq
☒	☐	Flow cytometry
☒	☐	MRI-based neuroimaging

Animals and other research organisms

Policy information about studies involving animals; ARRIVE guidelines recommended for reporting animal research, and Sex and Gender in Research

Laboratory animals	Wild type Zebrafish larvae (Danio rerio) of the WIK strain, 5 days post fertilization (dpf).

Wild animals	The animals used are wild type zebrafish larvae of the WIK strain, 5 days post fertilization (dpf).
Reporting on sex	Larvae were used before sex determination takes place. Hence all larvae are neither male or female, but still sex in-determinant.
Field-collected samples	The study did not involve field-collected samples.
Ethics oversight	Experiments on larvae until 5 dpf do not fall under animal welfare regulations. Husbandry and housing was approved by local authorities (Kantonales Veterinäramt TV4206).

Note that full information on the approval of the study protocol must also be provided in the manuscript.
